# Pathophysiology characterization of Alzheimer’s disease in South China’s aging population: for the Greater-Bay-Area Healthy Aging Brain Study (GHABS)

**DOI:** 10.1186/s13195-024-01458-z

**Published:** 2024-04-16

**Authors:** Zhen Liu, Dai Shi, Yue Cai, Anqi Li, Guoyu Lan, Pan Sun, Lin Liu, Yalin Zhu, Jie Yang, Yajing Zhou, Lizhi Guo, Laihong Zhang, Shuqing Deng, Shuda Chen, Xianfeng Yu, Xuhui Chen, Ruiyue Zhao, Qingyong Wang, Pengcheng Ran, Linsen Xu, Liemin Zhou, Kun Sun, Xinlu Wang, Qiyu Peng, Ying Han, Tengfei Guo

**Affiliations:** 1 Institute of Biomedical Engineering, Shenzhen Bay Laboratory, No.5 Kelian Road, Shenzhen, 518132 China; 2 Neurology Medicine Center, The Seventh Affiliated Hospital, Sun Yat-sen University, Shenzhen, 518000 China; 3 Department of Neurology, Xuanwu Hospital of Capital Medical University, Beijing, 100053 China; 4 Department of Neurology, Peking University Shenzhen Hospital, Shenzhen, 518000 China; 5 Department of Nuclear Medicine, The First Affiliated Hospital, Guangzhou Medical University, Guangzhou, 510120 China; 6 Department of Neurology, University of Chinese Academy of Sciences-Shenzhen Hospital, Shenzhen, 518107 China; 7 Department of Nuclear Medicine, Guangdong Hospital of Traditional Chinese Medicine, Guangzhou, 510120 China; 8 Department of Medical Imaging, University of Chinese Academy of Sciences-Shenzhen Hospital, Shenzhen, 518106 China; 9 Institute of Cancer Research, Shenzhen Bay Laboratory, Shenzhen, 518132 China; 10 School of Biomedical Engineering, Hainan University, Haikou, 570228 China; 11 Center of Alzheimer’s Disease, Beijing Institute for Brain Disorders, Beijing, 100053 China; 12 National Clinical Research Center for Geriatric Diseases, Beijing, 100053 China; 13 Institute of Biomedical Engineering, Peking University Shenzhen Graduate School, Shenzhen, 518055 China

**Keywords:** Alzheimer’s disease, Plasma biomarker, PET, MRI, Chinese aging cohort

## Abstract

**Introduction:**

The Guangdong-Hong Kong-Macao Greater-Bay-Area of South China has an 86 million population and faces a significant challenge of Alzheimer’s disease (AD). However, the characteristics and prevalence of AD in this area are still unclear due to the rarely available community-based neuroimaging AD cohort.

**Methods:**

Following the standard protocols of the Alzheimer’s Disease Neuroimaging Initiative, the Greater-Bay-Area Healthy Aging Brain Study (GHABS) was initiated in 2021. GHABS participants completed clinical assessments, plasma biomarkers, genotyping, magnetic resonance imaging (MRI), β-amyloid (Aβ) positron emission tomography (PET) imaging, and tau PET imaging. The GHABS cohort focuses on pathophysiology characterization and early AD detection in the Guangdong-Hong Kong-Macao Greater Bay Area. In this study, we analyzed plasma Aβ_42_/Aβ_40_ (A), p-Tau_181_ (T), neurofilament light, and GFAP by Simoa in 470 Chinese older adults, and 301, 195, and 70 had MRI, Aβ PET, and tau PET, respectively. Plasma biomarkers, Aβ PET, tau PET, hippocampal volume, and temporal-metaROI cortical thickness were compared between normal control (NC), subjective cognitive decline (SCD), mild cognitive impairment (MCI), and dementia groups, controlling for age, sex, and *APOE-ε4*. The prevalence of plasma A/T profiles and Aβ PET positivity were also determined in different diagnostic groups.

**Results:**

The aims, study design, data collection, and potential applications of GHABS are summarized. SCD individuals had significantly higher plasma p-Tau_181_ and plasma GFAP than the NC individuals. MCI and dementia patients showed more abnormal changes in all the plasma and neuroimaging biomarkers than NC and SCD individuals. The frequencies of plasma A+/T+ (NC; 5.9%, SCD: 8.2%, MCI: 25.3%, dementia: 64.9%) and Aβ PET positivity (NC: 25.6%, SCD: 22.5%, MCI: 47.7%, dementia: 89.3%) were reported.

**Discussion:**

The GHABS cohort may provide helpful guidance toward designing standard AD community cohorts in South China. This study, for the first time, reported the pathophysiology characterization of plasma biomarkers, Aβ PET, tau PET, hippocampal atrophy, and AD-signature cortical thinning, as well as the prevalence of Aβ PET positivity in the Guangdong-Hong Kong-Macao Greater Bay Area of China. These findings provide novel insights into understanding the characteristics of abnormal AD pathological changes in South China’s older population.

**Supplementary Information:**

The online version contains supplementary material available at 10.1186/s13195-024-01458-z.

## Background

Alzheimer’s dementia patients suffer from memory loss, cognitive dysfunction, behavioral abnormalities, and social disorders [1]. Alzheimer’s disease (AD) is the leading cause of dementia, accounting for 60–80% of all cases [2]. Extracellular β-amyloid (Aβ) plaques and neurofibrillary tau tangles are the two key hallmarks of AD [3]. AD patients have reduced Aβ_42_ concentrations in cerebrospinal fluid (CSF) [4] or plasma [5], elevated cortical Aβ plaques [6–8], CSF or plasma phosphorylated Tau (p-Tau) concentrations [9], and cortical tau tangles [10], which eventually result in synaptic loss [11–13], hippocampal atrophy [14], Temporal-metaROI cortical thinning [15], hypometabolism and cognitive decline [4, 9, 16]. Such abnormal changes in Aβ and tau can be detected by biomarkers or positron emission tomography (PET) imaging 15–20 years before the earliest clinical symptoms of AD [17–19]. According to the research framework proposed by the National Institute on Aging and Alzheimer’s Association in 2018 [20], cognitively unimpaired (CU) older adults with the evidence of Aβ pathology measured by either CSF Aβ biomarker [4] or Aβ PET imaging [6] are defined as preclinical AD. Moreover, around 30% of the CU individuals aged 70 and over are at the preclinical AD stage [21] and have a high risk of cognitive decline in the future [22, 23].

The prevalence of dementia and mild cognitive impairment (MCI) among older adults aged 60 and above in China is 6.0% and 15.5%, respectively. Among those cases, the majority (65%) is dementia or MCI due to AD, followed by vascular dementia (26.7%) and other dementias (8.3%) [24]. Additionally, the prevalence of preclinical AD defined by Aβ PET imaging among adults aged 60 or older was around 18% [21]. Approximately 260 million individuals are 60 years or older in China [25]. Among them, around 15 million, 39 million, and 47 million individuals are at the stage of dementia, MCI, and preclinical AD, respectively. China’s total expenditure on dementia treatment and nursing services will be $1.89 trillion around 2050 [26]. AD has become the fifth leading cause of death disease in China in 2019 [27]. The age-standardized prevalence and the age-standardized death rate of AD and related dementias were 788.3/100 000 and 23.3/100 000, separately, which were slightly higher than that of the global levels (682.5/100 000 and 22.9/100 000, separately) [27]. The high prevalence and vast population of AD severely challenge China.

Recently, three anti-Aβ drugs, including Aducanumab [28], Lecanemab [29], and Donanemab [30] showed positive results in phase 3 clinical trials. Consequently, early AD diagnosis and intervention are critical for preventing AD progression. However, the standardization of biomarker measurements, magnetic resonance imaging (MRI), and PET image scanning and processing of AD are not fully established yet in China. Therefore, the neuroscientists, neurologists, pathologists, radio-pharmacists, biomedical engineers, and biochemists in Guangdong-Hong Kong-Macao Greater-Bay-Area are working together to initiate the Greater-Bay-Area Healthy Aging Brain Study (GHABS), aiming to investigate the pathological features and progression patterns of AD, especially in asymptomatic stage of AD. GHABS participants will undergo clinical neuropsychological assessments, biospecimen sample collection, MRI imaging, and PET imaging. The GHABS project aims to: (1) explore the risk factors of Aβ and tau aggregation in the early stage of AD among China’s aging population; (2) determine the effect of Aβ and tau pathologies upon downstream neurodegeneration and cognitive decline in both Aβ negative (Aβ-) and Aβ positive (Aβ+) elderly adults; (3) identify novel approaches and techniques for early detection of AD and provides significant reference for the target brain region and appropriate time window for anti-AD treatments.

In this study, we first summarized the aims, study design, data collection, and potential applications of GHABS. Second, we compared the plasma Aβ_42_/Aβ_40_, plasma p-Tau_181_, plasma GFAP, plasma NfL, Aβ PET, tau PET, hippocampal volume, and temporal-MetaROI cortical thickness between normal control (NC), subjective cognitive decline (SCD), MCI, and dementia groups. Third, we also reported the prevalence of plasma A/T staging and Aβ PET positivity of different diagnostic groups in the Guangdong-Hong Kong-Macao Greater-Bay-Area of South China.

## Methods

### Study design

Shenzhen Bay Laboratory launched the community-based longitudinal cohort study GHABS (ClinicalTrials.gov ID: NCT06183658) in May 2021. The GHABS project was approved by the Shenzhen Bay Laboratory’s and the collaborated hospitals’ Ethical Committees. The flow chart of participant engagement in the GHABS is delineated in Supplementary Fig. 1. The GHABS participants were recruited via posters and lectures in the community and nursing homes. Each participant signed the written informed consent of the GHABS project before enrollment. The participants who met the inclusion and exclusion requirements were informed about the baseline and follow-up examinations. From 2021 to 2026, the GHABS cohort will recruit 1400 individuals aged 55 and older, including 1100 CU older adults, 200 MCI patients, and 100 dementia patients. The scheme for recruiting GHABS participants is illustrated in Supplemental Fig. 1.

**Fig. 1:**
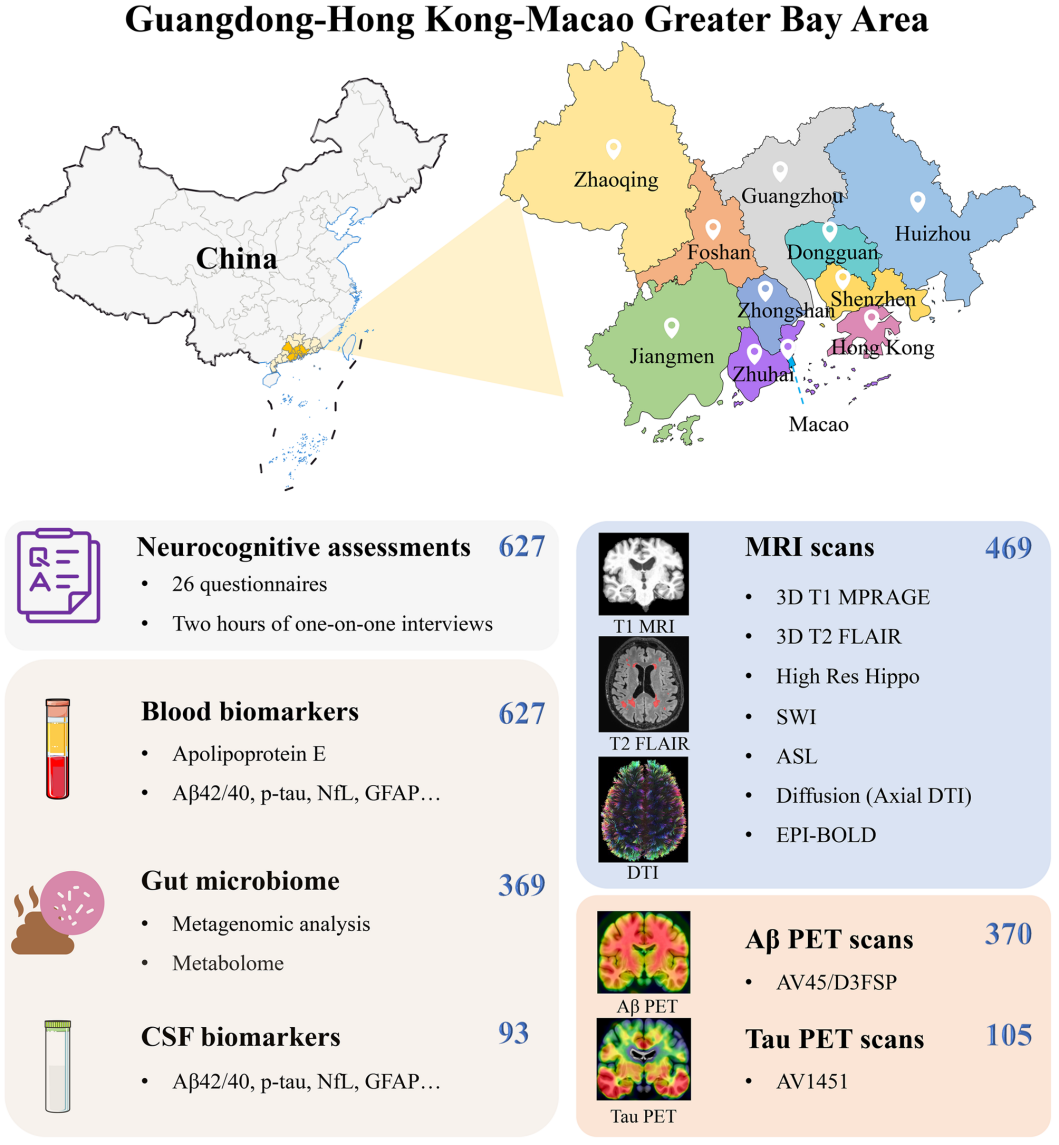
The current sample sizes of neurocognitive assessments, blood samples, CSF samples, stool samples, multimodal MRI scans, Aβ PET scans, and tau PET scans of the Greater-Bay-Area Healthy Aging Brain Study (By Sep 2023)

All the GHABS participants will undergo cognitive assessments, genetic screening, and blood sample collection. Some will have CSF collection, stool sample collection, MRI scanning, Aβ PET scanning, and tau PET scanning. All baseline examinations will be completed within three months. At follow-up, clinical assessments and blood sample collection will be conducted annually. CSF sample collection, MRI scan, Aβ PET scan, and tau PET scan are evaluated every two years. Six hundred twenty-seven participants have completed cognitive assessments and blood sample collection in the GHABS cohort by Sep 08, 2023 (Fig. 1). Among them, 26%, 44%, 19%, and 11% of the cohort were NC, SCD, MCI, and dementia, respectively. Additionally, 369 and 93 participants completed stool and CSF sample collection. So far, 469, 370, and 105 participants had MRI, Aβ PET, and tau PET imaging scans, respectively. This study identified 470 GHABS participants who simultaneously completed cognitive assessments, plasma Aβ_42_/Aβ_40_, p-Tau_181_, NfL, and GFAP data measured by the Simoa platform. Among them, 119, 207, 87, and 57 were NC, SCD, MCI, and dementia. Moreover, 301, 195, and 70 individuals had concurrent MRI, ^18^F-D3-FSP Aβ PET, and FTP tau PET scans.

### Inclusion and exclusion criteria

Briefly, the inclusion criteria of GHABS are as follows: (1) adults between the ages of 55 and 90 and speak Mandarin fluently [31]). Notably, individuals below 60 years old are required to have a family dementia history and meet the criteria of subjective cognitive decline (SCD), while people with family history of autosomal dominant or other familial AD are not limited by age; (2) the score on the Geriatric Depression Scale (GDS) is less than 6 points; (3) visual and auditory acuity is sufficient for neuropsychological testing (Including normal corrected vision and hearing); (4) female participants are not pregnant, lactating, or of childbearing potential (that is, women must be two years postmenopausal or surgically sterile); (5) a modified version of the Hachinski Ischemic scores less than or equal to 4; (6) have completed primary school (6 years of education) or have good work experience (sufficient to rule out mental retardation). Individuals with an infection, infarction, or other focal lesions or multiple lacunes or lacunes in critical memory structures and who do not meet the MRI scanning requirements are excluded from the GHABS study. More details of the inclusion and exclusion criteria can be found in the supplemental materials.

### Cognitive assessments

The cognitive profiles are assessed via a series of cognitive ability tests, including the Alzheimer’s Disease Assessment Scale–Cognitive Subscale (ADAS-Cog), Logical Memory Test I & II (the Chinese version), Mini-mental State Examination (MMSE, the Chinese version), Montreal Cognitive Assessment Basic (MoCA-Basic), Shape Trail Test (STT), Clock Drawing Test (CDT), Auditory Verbal Learning Test (AVLT), Symbol Digit Modalities Test (SDMT), Digit Span Test (DST), Animal Verbal Fluency Test (AFT), Cognitive Change Index (CCI), SCD. Besides, functional and behavioral tests were also executed, including the Hachinski Ischemic Score (HIS), Clinical Dementia Rating (CDR), Neuropsychiatric Inventory (NPI), the GDS, Functional Activities Questionnaire (FAQ), Activity of Daily Living Scale (ADL) [32–34], measurement of everyday cognition (Ecog), Pittsburgh sleep quality index (PSQI), REM sleep behavior disorder screening questionnaire (RBDSQ), Epworth Sleepiness Scale (ESS).

The participants were classified as NC, MCI, or dementia due to AD following the standard protocol of ADNI cohort [35]. We further define some CU individuals as SCD following the research criteria proposed by Jessen and colleagues in 2014 [36]. Several cognitive assessments were used for clinical diagnosis. The normal performance of these assessments was defined based on education or age. The MMSE used cutoff scores: >17 for participants without education, > 20 for 1–6 years of education, and > 24 for more than six years of education. The delayed recall of logical Memory test used cutoff scores as follows: ≥3 for 0–7 years, ≥5 for 8–15 years, and ≥9 for 16 years of education [37]. As for the ADL, < 23 is normal for participants under 75 years old, while < 25 is for 75 years and older.

CU participants were normal in MMSE, logical Memory recall, and ADL, and their CDR score was 0. Among CU individuals, the presence of the following symptoms as having SCD: (1) Self-experienced persistent decline in cognitive capacity in comparison with a previously normal status and unrelated to an acute event; (2) Normal age-adjusted, gender-adjusted and education-adjusted performance on standardized cognitive tests. CU individuals without SCD were defined as the NC group. Participants with MCI had unimpaired MMSE, while they showed impairment in the logical Memory test [38]. Their CDR score was 0.5, with a mandatory requirement of the memory box score being 0.5 or greater, but ADL was normal. Dementia due to AD was abnormal in MMSE, logical memory, and ADL. The CDR score was 0.5 or greater.

### Biospecimen collection

Volunteers fasted for one night the day before (not less than 6 h), and blood was drawn in the morning of the next day. The venous blood of the volunteers is drawn into two 10 ml EDTA blood collection tubes and gently inverted and mixed 10–12 times to ensure that the blood and anticoagulant are thoroughly mixed. The mixed blood was placed in an incubator at 4 °C and shipped back to the laboratory within 4 h for subsequent analysis. The blood is centrifuged at 1600 g for 10 min in a refrigerated centrifuge at 4 °C. The upper plasma layer is transferred to several 2 ml centrifuge tubes using a sterile RNase-free pipette tip. To obtain more pure plasma, the separated plasma is centrifuged again at 16,000 g for 10 min at 4 °C, and then the supernatant is aliquoted into several 0.5 ml centrifuge tubes with labels, each with either 100–200 µl blood plasma. The aliquots are stored in a -80 °C refrigerator for subsequent analysis. After the whole blood is centrifuged in the first step, the buffy coat in the middle layer is gently transferred to the 2640 medium. Then, after density gradient centrifugation, erythrocyte lysis, and centrifugation steps, the isolated peripheral blood mononuclear cell sample is transferred to a 2 ml RNase-free centrifuge tubes and stored in a gradient-cooled freezer box at -80 °C for subsequent analysis. Samples will be used for genomic analysis (including whole genome sequencing and other analyses).

Before collecting the CSF sample, the volunteer must fast for one night (at least 6 h), and the lumbar spinal fluid is performed on an empty stomach the following day. Lumbar puncture is performed strictly with clinical standards, and about 5 ml of CSF is collected. The collected CSF is sent to Shenzhen Bay Laboratory within 2 h for biomarker analysis. CSF sample is quickly divided into 1.5 ml low protein adsorption centrifuge tubes. Afterward, they will be stored in a -80 °C refrigerator for subsequent analysis.

Fecal samples are collected on-site or at home after the volunteers’ consent is obtained. The volunteer stool samples are labeled, sub-packaged, and frozen in a -80 °C refrigerator. Fecal samples are used for 16 S rDNA, metagenomic, metagenome, and metabolome detection of intestinal microorganisms.

### CSF and plasma biomarkers measurement

The concentrations of Aβ_40_, Aβ_42_, NfL, GFAP, SNAP25, p-Tau_181_, and p-Tau_217_ in CSF and plasma are detected using commercial Simoa® NEUROLOGY 4-PLEX E (N4PE, cat: 103,670), SNAP-25 (cat: 103,575), pTau-181 (cat: 104,111), and pTau-217 kit in Simoa HD-X Analyzer™ (Quanterix Corp.). The concentrations of YKL40 in CSF and plasma are measured using a commercial Human YKL-40 Assay (cat: K151VLK) in MESO SECTOR S 600MM (Meso Scale Diagnostics, LLC.). The concentrations of sTREM2 in CSF and plasma are also measured by the MSD platform using an in-house immunoassay as previously described [39]. Briefly, streptavidin-coated plates are blocked overnight in PBS containing 3% bovine serum albumin (BSA) and 0.05% Tween-20 before incubating with the biotinylated capture antibody (0.25 µg/mL, cat: BAF1828) for 1 h. After washing with PBS containing 0.05% Tween-20, the plates are incubated with CSF, and plasma samples diluted in PBS containing 0.25% BSA, 0.05% Tween-20, and Protease Inhibitor Cocktail (cat: P8340) for 2 h. Recombinant human TREM2 protein (SinoBiological, cat. 11,084-H08H) is used as a standard (62.5 to 8000 pg/mL). The plates are rewashed and incubated with the detector antibody (1 µg/mL, cat: sc373828) for 1 h, followed by incubation with the MSD SULFO-tag conjugated secondary antibody (0.5 µg/mL, cat: R32AC). Finally, the electrochemical signal is developed by adding 2x MSD Read buffer T (cat: R-92TC), and the MSD SECTOR S 600MM measures the electro chemiluminescent signal.

The concentrations of PDGFR-β and GAP43 in CSF and plasma are measured by commercial Human PDGFR-β (R&D, Catalog Number: DYC385) and GAP43 (Abbexa) ELISA kits. For neurogranin, an in-house sandwich ELISA is developed combining the mouse monoclonal antibodies Ng22 and Ng2 (Abcam). Nunc maxisorp 96-well microliter plates (Thermo Fisher Scientific) are coated with the mouse anti-human neurogranin (Ng22, 3 µg/mL) in bicarbonate buffer pH 9.6 overnight. After washing with PBS containing 0.05% Tween-20, the plates are blocked in PBS containing 1% BSA and 0.05% Tween-20 for 1 h, followed by incubation with CSF and plasma samples overnight. Recombinant full-length human neurogranin protein is used as a standard (25 to 3200 pg/ml for CSF, 55 to 40,000 pg/mL for plasma). After additional washes, the plates are incubated with the biotinylated mouse anti-human neurogranin (Ng2, 2.7 µg/mL) for 1 h, followed by incubation with a Streptavidin-HRP (R&D Systems) for 30 min. After additional washes, the plates are incubated with Substrate Reagent (TMB, R&D Systems) for 10 min in the dark. The color reaction is stopped by adding 0.2 M H_2_SO_4,_ and the absorbance is read at 450 nm (650 nm as a reference value).

All samples were analyzed randomly and double-blindly to avoid bias because of the effect of inter-assay variability on specific patient groups. For all biomarker measurements, the samples in the first plate were tested in duplicate, and subsequent samples were tested in single. A pooled sample was generated as a reference, aliquoted and stored at -80 °C. In each measurement, the reference sample was tested in duplicate to compare the detection variability. The mean intra-assay and inter-assay coefficient of variation (CV) was controlled within 20%.

### MRI image acquisition

All the MRI scanning sequences will be conducted following the standard ADNI protocol here. The MRI image data is collected on 3.0T scanners, and the scanning parameters vary slightly depending on the specifics of scanners from various clinical centers. A series of sequences are applied for head imaging of each volunteer, including3D T1 MPRAGE, 3D T2 FLAIR (Fluid-Attenuated Inversion Recovery), High Res Hippo (High Resolution Hippocampus), SWI (Susceptibility Weighted Imaging), ASL (Arterial spin labeling), Diffusion (Axial DTI), Field Mapping, EPI-BOLD. Each sequence and parameters of MRI scanning are described as follows:

3D MPRAGE scan parameters: field of view (FOV) = 208 × 240 × 256 mm^3^; matrix = 208 × 240 × 256 (resolution 1 × 1 × 1 mm^3^); imaging plane: sagittal; TR = 2300 ms; TE = minimum TE value of the system; TI = 900 ms; FA = 7°; 2-fold acceleration in the phase-encoding direction; scan time 6 min 11 s. Purpose: (1) To provide high-resolution anatomical structure templates for low-resolution functional imaging (PET, EPI images, etc.); (2) To calculate the brain tissue volume and cortical thickness of the subjects, and to measure brain atrophy. Note that the whole brain was covered to avoid aliasing artifacts.

3D FLAIR scanning parameters: FOV = 256 × 256 × 260 mm; matrix = 214 × 256 × 160 (resolution 1.2 × 1 × 1 mm); imaging plane: sagittal; TR = 4800 ms; TE = 119 ms; TI = 1650 ms; 3-fold acceleration in the phase-encoding direction; scan time 6 min 48 s. Purpose: To perform white matter hyperintensity (WMH) segmentation and to evaluate white matter lesions, including infarction and other pathological features.

High Res Hippo scanning parameters: FOV = 175 × 60 × 175 mm^3^; matrix = 449 × 30 × 449 (resolution 0.39 × 2 × 0.39 mm^3^); Oblique scan; layer direction perpendicular to the long axis of the hippocampus; TR = 8020 ms; TE = 50 ms; 2-fold acceleration in the phase-encoding direction; scan time 5 min 36s. Purpose: To perform hippocampal segmentation. Note: (1) the layer direction is perpendicular to the long axis of the hippocampus; (2) the imaging FOV should cover the skull, the hippocampal head, and the hippocampus tail.

SWI scanning parameters: FOV = 220 × 220 × 130 mm^3^; matrix = 368 × 368 × 130 (resolution 0.6 × 0.6 × 1 mm^3^); imaging plane: transverse; TR = 55 ms; 6 echoes, with the TE of 1st echo = 7.7 ms and the delta TE between echoes = 7.0 ms; 2-fold acceleration in the phase-encoding direction; scan time 6 min 10 s. Purpose: (1) To evaluate cerebral microbleeds; (2) To obtain the quantitative susceptibility map (QSM).

ASL scanning parameters: 3D pseudo-continuous ASL (pCASL) series; FOV = 240 × 240 × 160 mm^3^; matrix = 96 × 96 × 40 (resolution: 2.5 × 2.5 × 4 mm^3^); transverse positioning; TR = 4250 ms; TE = 9.0 ms; Label duration = 1800 ms; Post label delay (PLD) = 2000 ms; 2-fold acceleration in the phase-encoding direction; axial acquisition direction: fat chemical shift toward the posterior direction; a proton density image was also acquired within the same sequence to quantify cerebral blood flow (CBF) from the ASL series. The scanning time is 6 min 68 s. Purpose: To measure whole brain perfusion and to calculate CBF. Notes: (1) the labeling plane was placed 20 mm inferior to the lower edge of the imaging volume; (2) cover the cerebellum; (3) keep eyes open, not to think of anything in particular, and remain still during the scan.

Diffusion scanning parameters: FOV = 232 × 232 × 176 mm^3^; matrix = 116 × 116 × 88 (resolution: 2 × 2 × 2 mm^3^); transverse position; TR = 3300 ms; TE = 71 ms; multi-b value acquisition: b = 0, 1000, 2000 s/mm^2^ (in total 112 diffusion directions); 2-fold acceleration in the phase-encoding direction and multiband factor = 3; axial acquisition direction: fat chemical shift toward the posterior direction; scan time 11 min 16 s. 8 averages of b = 0 DTI images with fat chemical shift toward the anterior direction of the axial acquisition direction were also scanned for the geometry distortion correction. Purpose: (1) To evaluate the diffusion parameters of white matter fibers in the brain, such as anisotropy FA, AD, MD, and other parameters; (2) To track white matter fibers in the brain and evaluate the structural connection of white matter fibers. Note to cover the whole brain.

Field mapping scanning parameters: FOV = 220 × 220 × 160 mm^3^; matrix = 88 × 88 × 64 (resolution: 2.5 × 2.5 × 2.5 mm^3^); imaging plane: transverse; TR = 400 ms; TE1 = 4.92 ms, TE2 = 7.38 ms. Purpose: Used for geometry distortion correction for EPI series.

Echo-planar imaging (EPI) - Blood oxygen level-dependent (BOLD) scanning parameters: FOV = 220 × 220 × 160 mm^3^; matrix = 88 × 88 × 64 (resolution: 2.5 × 2.5 × 2.5 mm^3^); imaging plane: transverse; TR = 600 ms; TE = 30 ms; FA = 53° (Ernst angle with best BOLD contrast); Echo spacing = 0.49 ms; 2-fold acceleration in the phase-encoding direction and multiband factor = 4; axial acquisition direction: fat chemical shift toward the posterior direction; scan time 10 min. Purpose: to assess the functional connectivity between any pair of brain regions. Notes that participants were instructed to keep their eyes open, not to think of anything in particular, and to remain still during the scan.

### PET image acquisition

The Aβ PET radiotracer [^18^F]-florbetapir (FBP) [40] or [^18^F]D3FSP (FSP) [41] and tau PET radiotracer [^18^F]-flortaucipir (FTP) [42] are used for PET imaging. The data acquisition is performed on either a GE Discovery™ MI Gen 2 PET/CT scanner or a Siemens Biograph™ TruePoint™ TrueV PET/CT scanner. The spatial resolution of each PET scanner is quantified with PET imaging of a Hoffman phantom. For the Aβ PET imaging, the subjects are injected with either [^18^F]-florbetapir or [^18^F]-D3FSP intravenously at 370 MBq (10 mCi ± 10%), rested for 45 min and prepared for the scanning. PET/CT imaging is performed 50 min after injection, and the PET acquisition time is 20 min. For the tau PET imaging, the participants are injected with [^18^F]-flortaucipir intravenously at 370 MBq (10 mCi ± 10%), rested for 75 min, and prepared for imaging. The dynamic acquisition of [^18^F]-flortaucipir tau PET data is completed 80–100 min after the radiotracer administration.

A dedicated head scanning procedure covering the whole brain from vertex to cerebellum is used for imaging. A diagnostic dose CT scan of the brain is acquired beforehand for attenuation correction and fusion localization of PET images. The PET scans are acquired using 3D list mode on the GE Discovery MI and Siemens BioGraph TruePoint scanners in two sites. For the GE scanner, the FOV is 256 mm×256 mm×220 mm, the scanning matrix is 336 × 336 × 109, and the voxel size is 1.02 mm×1.02 mm×2.03 mm. For the Siemens scanner, FOV is 256 mm×256 mm×198 mm, the scanning matrix is 192 × 192 × 71, and the voxel size is 1.33 mm×1.33 mm×2.79 mm. All the correction options were selected for both scanners, and no filter or smooth was used during the reconstruction. A reconstruction offset is applied to ensure that the head is entirely in the field of view within the plane. Finally, 4 frames of dynamic images are generated according to 5 min/frame segmentation, with each PET scan corresponding to a 20-minute PET image.

### MRI and PET imaging analysis

The structural MRI images are segmented into different cortical and subcortical regions of interest (ROI) in Freesurfer (V7.2.0). The residual hippocampal volume (rHCV) is calculated using the hippocampal volume of both hemispheres and adjusted using the estimated total intracranial volume as we described previously [14]. In addition, the cortical thickness of AD-signature atrophy brain regions is obtained by calculating the surface area-weighted average thickness of the bilateral entorhinal, fusiform, inferior temporal, and middle temporal cortices [43].

**Fig. 2:**
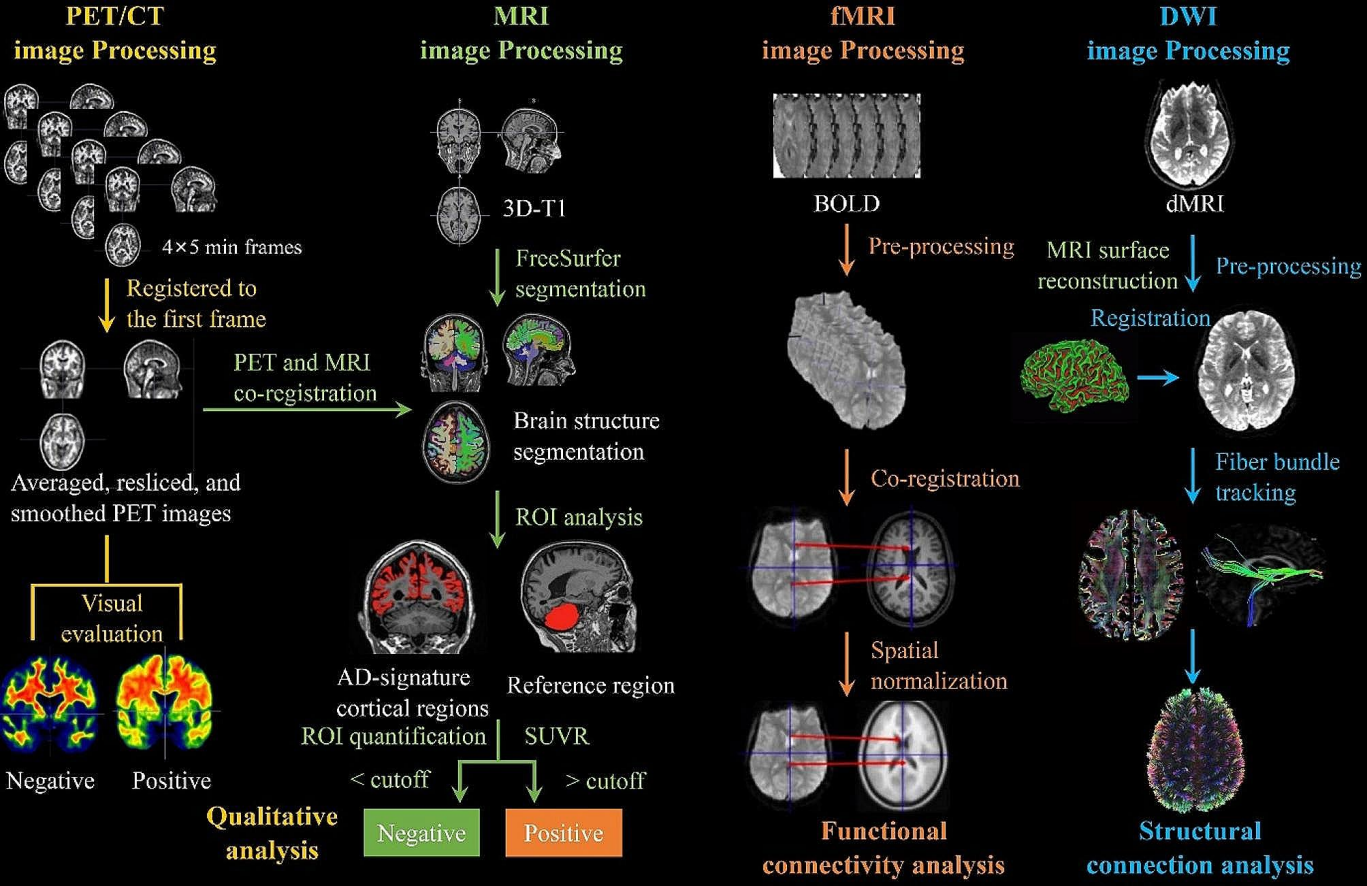
The image processing pipelines of PET, structural MRI, functional MRI (fMRI), and diffusion MRI data of the Greater-Bay-Area Healthy Aging Brain Study

As shown in Fig. 2, the PET images are preprocessed with the following steps before further analysis: (1) co-registering the 2nd, 3rd, and 4th frames to the 1st frame, respectively; (2) averaging the four frames into one averaged frame; (3) the averaged frame resliced into a standard AC-PC space (anterior commissure-posterior commissure) with image size = 160 × 160 × 128, voxel dimension = 1.5 mm×1.5 mm×1.5 mm; (4) this standardized image then served as a reference imaging, and each original individual frame was resliced to this reference imaging; (5) smoothing to a uniform 6 mm resolution using the Gaussian kernel function, and the full-width at half maximum (FWHM) of each site was determined from the Hoffman phantom [^18^F]FDG-PET imaging (As a reference for mitigating between-scanner differences in multi-center PET scans). The PET and MRI images are processed using in-house Matlab algorithms, as shown in Fig. 2. The PET images are co-registered with their corresponding structural MRI images in SPM12 (Statistical Parametric Mapping). Sixty-eight Freesurfer-defined cortical ROIs obtained from MRI segmentation extract regional FSP, FBP, and FTP measurements from the co-registered PET images.

The FSP and FBP standardized uptake value ratio (SUVR) of AD summary cortical regions (posterior cingulate cortex, precuneus, frontal lobe, parietal lobe, and lateral temporal) are obtained by dividing the radiotracer uptake value of AD typical brain regions by that in the whole cerebellum [44]. In the present study, we only presented FSP Aβ PET data. For the [^18^F]-flortaucipir images, FTP SUVR of 68 FreeSurfer-defined ROIs are calculated by normalizing the [^18^F]-flortaucipir value to the value of the inferior cerebellar cortex [45]. The FTP SUVR of the AD Temporal-MetaROI [43] (entorhinal cortex, parahippocampal gyrus, fusiform, amygdala, inferior temporal and middle temporal brain regions) is used to evaluate cortical tau deposition.

The general process of resting-state fMRI data preprocessing and brain functional connectivity construction are outlined in the third column of Fig. 2 and described in detail in the Supplemental Materials. The general process of diffusion MRI processing are outlined in the fourth column of Fig. 2 and described in detail in the Supplemental Materials. Diffusion-weighted data are denoised and corrected for Gibbs ring using Mrtrix3 (V3.0.3) [46], and then corrections were applied for head motion, eddy current, and EPI susceptibility distortion using FSL (V6.0.3) [47]. 3D pCASL imaging was employed to calculate the CBF map (Supplemental Fig. 3). The WMH segmentation was processed using a custom pipeline developed by our lab based on the T2 FLAIR images (Supplemental Fig. 4). More details of fMRI, dMRI, WMH, and pCASL can be found in Supplementary Material.

### Cutoffs of plasma Aβ_42_/Aβ_40_, and plasma p-Tau_181_, and FSP Aβ PET

We used Gaussian mixed model analysis to estimate two Gaussian distributions of low Aβ and high Aβ (Supplemental Fig. 5) for COMPOSITE FSP SUVR to define an unsupervised threshold, which corresponds to a 90% probability of belonging to the high Aβ distribution. The thresholds of FSP SUVR were defined as COMPOSITE SUVR≥0.76 (Supplemental Fig. 5). The receiver operating characteristic (ROC) curve analysis using the Youden index classifying 154 Aβ- cognitively unimpaired (CU) participants and 69 Aβ + cognitively impaired (CI) individuals (MCI or dementia) as the endpoint to define the cutoff ≤ 0.0609 for plasma Aβ_42_/Aβ_40_ ratio (Supplemental Figs. 6–7). Similarly, the ROC analysis using the Youden index classifying 143 Aβ- CU participants and 67 Aβ + CI participants as the endpoint to define the cutoff ≥ 2.418 for plasma p-Tau_181_ (Supplemental Figs. 8–9). The cohort was divided into A-/T-, A-/T+, A+/T-, and A+/T + according to the plasma Aβ_42_/Aβ_40_ ratio and plasma p-Tau_181_ thresholds.

### Statistical analysis

All the statistical analyses were conducted using R (v4.3.0, The R Foundation for Statistical Computing). The normal distribution of the data in this study was determined using the Shapiro-Wilk test. Plasma p-Tau_181_, plasma GFAP, and plasma NfL were log_10_ transferred before the following analysis to meet the normal distribution. We used a two-tailed Mann-Whitney U test and Fisher’s exact test to compare the continuous and categorical characteristics at baseline between different diagnosis groups, respectively. Data were presented as median (interquartile range, IQR) or No. (%) unless otherwise noted. Generalized linear models were used to compare plasma biomarkers, COMPOSITE FSP Aβ SUVR, temporal-metaROI FTP SUVR, rHCV, and temporal-metaROI cortical thickness between different clinical groups, controlling for age, sex, and *APOE-ε4*. Subsequently, we investigated the frequency of different A/T profiles defined by plasma biomarkers (A: plasma Aβ_42_/Aβ_40_, T: plasma p-Tau_181_) and Aβ PET positivity among NC, SCD, MCI, and dementia groups.

## Results

### Demographics of participants

The demographic characteristics of participants included in this study are summarized in Table 1. At baseline, MCI and dementia individuals had older ages, higher percentages of *APOE-ε4* carriers, shorter duration of education, and lower MoCA and MMSE scores than NC and SCD individuals. The dementia group also had lower MoCA and MMSE scores than the MCI group. The SCD group had more females than the MCI and dementia groups. Additionally, 443 participants had risk factors records, including the medical history of hypertension, hyperlipidemia, and diabetes, as well as the assessments of PSQI and GDS. SCD individuals had higher percentage of hyperlipidemia compared to the NC and MCI groups. SCD and MCI individuals had worse sleep state than NC and dementia individuals, while dementia individuals showed better sleep state than NC individuals. MCI and dementia individuals had higher depression scores than SCD and NC individuals, whereas SCD individuals showed higher depression scores than NC individuals. The demographic data of the GHABS cohort by cities are summarized in Supplemental Tables 2 and have been compared with the corresponding demographcis from the literature, as presented in Supplemental Table 3 [48, 49].

**Table 1:** Demographics of Greater-Bay-Area Healthy Aging Brain Study (GHABS) participants included in this study

	NC	SCD	MCI	Dementia
No., %	119, 25.3%	207, 44.0%	87, 18.5%	57, 12.1%
Age, years	66(9.2,55–89)	66(7.9,55–86)	^a, b^69(10,58–89)	^a, b^73(12.6,57–86)
APOE-ε4 (No., %)	24, 20.2%	36, 17.4%	^a, b^35, 40.2%	^a, b^27, 47.4%
Female (No., %)	72, 60.5%	^a, c, d^154, 74.4%	48, 55.2%	31, 54.4%
Education, years	13(4.0,5–20)	14(4.3,2–24)	^a, b^12(6.0,2–18)	^a, b^11(4.0,0–18)
MoCA	27 (3)	26 (4)	^a, b^22 (5)	^a, b, c^12 (7)
MMSE	29 (3)	29 (3)	^a, b^26 (4)	^a, b, c^18 (8)
**Participants with risk factors (*n*** = **443)**				
No., %	115, 26.0%	197, 44.5%	79, 17.8%	52, 11.7%
Hypertension (No., %)	24, 20.2%	41, 19.8%	18, 20.7%	8, 14.0%
Diabetes (No., %)	10, 8.4%	18, 8.7%	7, 8.0%	7, 12.3%
Hyperlipidemia (No., %)	21, 17.6%	^a^65, 31.4%	^b^10, 11.5%	7, 12.3%
PSQI	5.5 (4)	^a, d^7.0 (5)	^a, d^6.0 (6)	^a, b, c^4.0 (4.3)
GDS	2 (2)	^a, c, d^2 (3)	^a, b^3 (4)	^a, b^3 (4)
**Participants with MRI image data (*n*** = **301)**				
No., %	73, 24.3%	132, 43.9%	57, 18.9%	39, 13.0%
Age, years	66(8.9,55–89)	67(8.0,55–86)	^a^69(9.0,58–89)	^a, b^73(13.3,57–85)
APOE-ε4 (No., %)	19, 26.0%	24, 18.2%	^b^22, 38.6%	^a, b^20, 51.3%
Female (No., %)	43, 58.9%	94, 71.2%	33, 57.9%	23, 59.0%
MoCA	26 (3)	26 (3)	^a, b^22 (5)	^a, b, f^12 (7.5)
MMSE	29 (2)	29 (3)	^a, b^26 (3)	^a, b, f^18 (9)
**Participants with Aβ PET image data (*n*** = **195)**				
No., %	43, 22.0%	80, 41.0%	44, 22.6%	28, 14.4%
Age, years	66(8.6,55–89)	67(7.4,55–81)	^a, b^69(9.1,58–89)	^a, b^73(12.7,58–85)
APOE-ε4 (No., %)	13, 30.2%	12, 15.0%	^b^18, 40.9%	^b^15, 53.6%
Female (No., %)	24, 55.8%	52, 65.0%	24, 54.5%	17, 60.7%
MoCA	26 (4)	26 (3)	^a, b^23 (5)	^a, b, e^13.5 (10.5)
MMSE	28 (2)	29 (3)	^a, b^27 (3)	^a, b, e^18 (8.25)
**Participants with tau PET image data (*n*** = **70)**				
No., %	18, 25.7%	23, 32.9%	18, 25.7%	11, 15.7%
Age, years	65(12.7,55–89)	65(6.0,57–80)	70(8.5,58–78)	62(10.4,58–80)
APOE-ε4 (No., %)	7, 38.9%	11, 47.8%	11, 61.1%	7, 63.6%
Female (No., %)	8, 44.4%	17, 73.9%	9, 50.0%	6, 54.5%
MoCA	26.5 (4)	26 (5.5)	^a, b^22 (5.75)	^a, b, f^12 (8.5)
MMSE	28.5 (3)	28 (3)	^b^26 (3.75)	^a, b, f^13 (9)

*Note*^a, b, c, d^ indicates significantly different from NC, SCD, MCI, and dementia groups respectively

*Abbreviations* Aβ = β-amyloid; NC = Normal control; SCD = subjective cognitive decline; MCI = mild cognitive impairment; PSQI = Pittsburgh Sleep Quality Index; GDS = Geriatric Depression Scale

### Comparisons of plasma biomarkers and neuroimages among different clinical stages

**Fig. 3:**
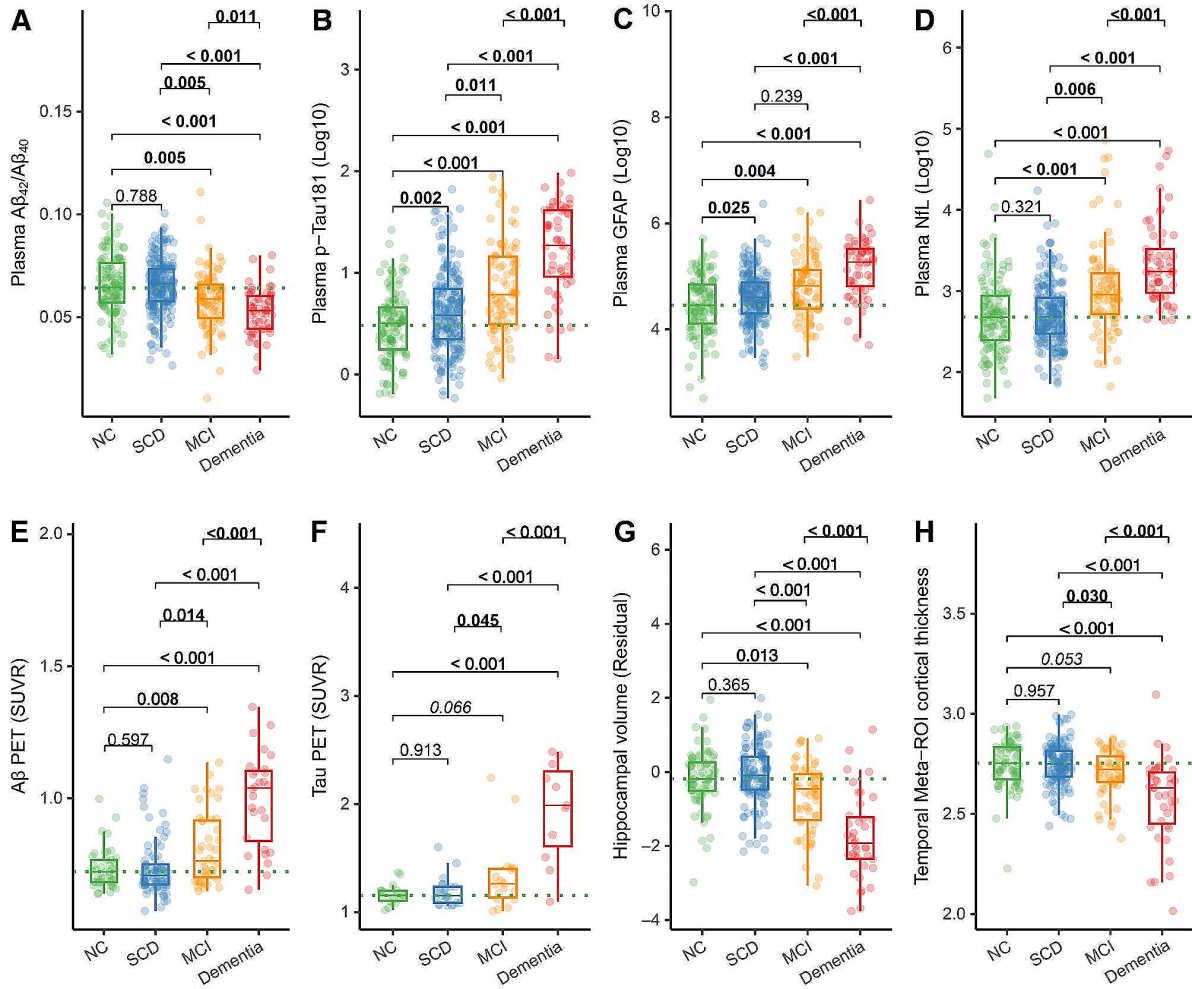
Comparisons of plasma biomarkers and neuroimaging between different clinical groups. Comparisons of (**A**) plasma Aβ_42_/Aβ_40_, (**B**) plasma p-Tau_181_, (**C**) plasma GFAP, (**D**) plasma NfL, (**E**) Aβ PET, (**F**) tau PET, (**G**) hippocampal volume and (**H**) temporal-MetaROI cortical thickness between NC, SCD, MCI, and dementia groups. Plasma p-Tau_181_, plasma GFAP, and plasma NfL were log_10_ transferred. The mean intra-assay and inter-assay coefficient of variation (CV)s were respectively 12% and 17% for plasma Aβ_42_, 9% and 17% for plasma Aβ_40_, 7% and 9% for plasma NfL, 5% and 14% for plasma GFAP, and 10% and 13% for plasma p-Tau_181_

Compared to the NC group, the SCD group had higher plasma p-Tau_181_ (standardized β (β_std_) = 0.304[95% confidence interval (ci), 0.113, 0.494], *p* = 0.002) and plasma GFAP (β_std_ = 0.225[95% ci, 0.029, 0.420], *p* = 0.025), and the MCI group had lower plasma Aβ_42_/Aβ_40_ (β_std_ = -0.392[95% ci, -0.663, -0.121], *p* = 0.005), higher plasma p-Tau_181_ (β_std_ = 0.594[95% ci, 0.352, 0.836], *p* < 0.001), plasma GFAP (β_std_ = 0.363[95% ci, 0.114, 0.611], *p* = 0.004), plasma NfL (β_std_ = 0.388[95% ci, 0.161, 0.615], *p* < 0.001), COMPOSITE Aβ PET SUVR (β_std_ = 0.452[95% ci, 0.117, 0.788], *p* = 0.008), and temporal-metaROI FTP SUVR (β_std_ = 0.420[95% ci, -0.028, 0.867], *p* = 0.066), and more decreases in rHCV (β_std_ = -0.347[95% ci, -0.622, -0.072], *p* = 0.013) (Fig. 3).

The MCI group showed lower plasma Aβ_42_ (Supplemental Fig. 10, β_std_ = -0.365[95% ci, -0.619, -0.111], *p* = 0.014), plasma Aβ_42_/Aβ_40_ (Fig. 3A, β_std_ = -0.363[95% ci, -0.614, -0.112], *p* = 0.005), rHCV (Fig. 3G, β_std_ = -0.451[95% ci, -0.703, -0.200], *p* < 0.001), and temporal-metaROI cortical thickness (Fig. 3H, β_std_ = -0.315[95% ci, -0.600, -0.030], *p* = 0.030), and higher plasma p-Tau_181_ (Fig. 3B, β_std_ = 0.290[95% ci, 0.066, 0.514], *p* = 0.011), plasma NfL (Fig. 3D, β_std_ = 0.297[95% ci, 0.087, 0.508], *p* = 0.006), COMPOSITE Aβ PET SUVR (Fig. 3E, β_std_ = 0.373[95% ci, 0.074, 0.673], *p* = 0.014), temporal-metaROI FTP SUVR (Fig. 3F, β_std_ = 0.444[95% ci, 0.011, 0.878], *p* = 0.045) than the SCD group.

The dementia patients had significant abnormal alternations (*p* ≤ 0.011) in all the plasma biomarkers, and Aβ PET, tau PET, rHCV, and temporal-metaROI cortical thickness than the other three groups (Fig. 3). Besides, the dementia group had lower plasma Aβ_42_ compared to the NC group (β_std_ = -0.556[95% ci, -0.876, -0.237], *p* = 0.002) and SCD group (β_std_ = -0.597[95% ci, -0.897, -0.297], *p* < 0.001) as shown in Supplemental Fig. 9.

### Frequency of plasma A/T and Aβ PET positivity among different clinical stages

**Fig. 4:**
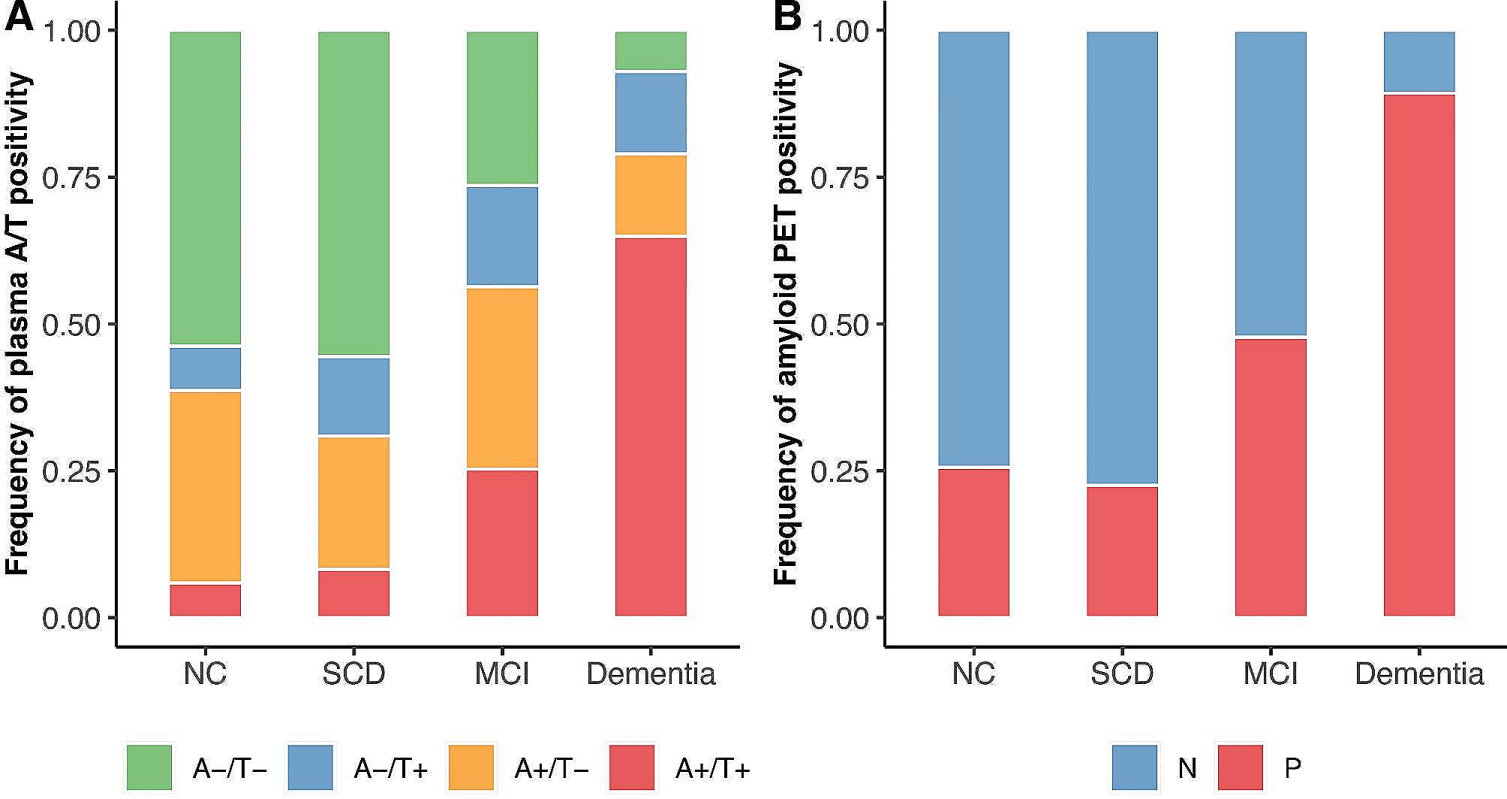
The frequency of plasma A/T profiles and Aβ PET positivity between different clinical groups. Comparisons of (**A**) A/T profiles defined by plasma Aβ_42_/Aβ_40_ and plasma p-Tau_181_, and (**B**) Aβ PET positivity between NC, SCD, MCI, and Dementia groups

The frequencies of A-/T- (NC; 53.8%, SCD: 55.6%, MCI: 26.4%, Dementia: 7.0%), A-/T+ (NC; 7.5%, SCD: 13.5%, MCI: 17.2%, Dementia: 14.0%), A+/T- (NC; 32.8%, SCD: 22.7%, MCI: 31.0%, Dementia: 14.0%), and A+/T+ (NC; 5.9%, SCD: 8.2%, MCI: 25.3%, Dementia: 64.9%) profiles were illustrated in Fig. 4A. The frequencies of Aβ PET positivity (NC: 25.6%, SCD: 22.5%, MCI: 47.7%, Dementia: 89.3%) were illustrated in Fig. 4B.

**Fig. 5:**
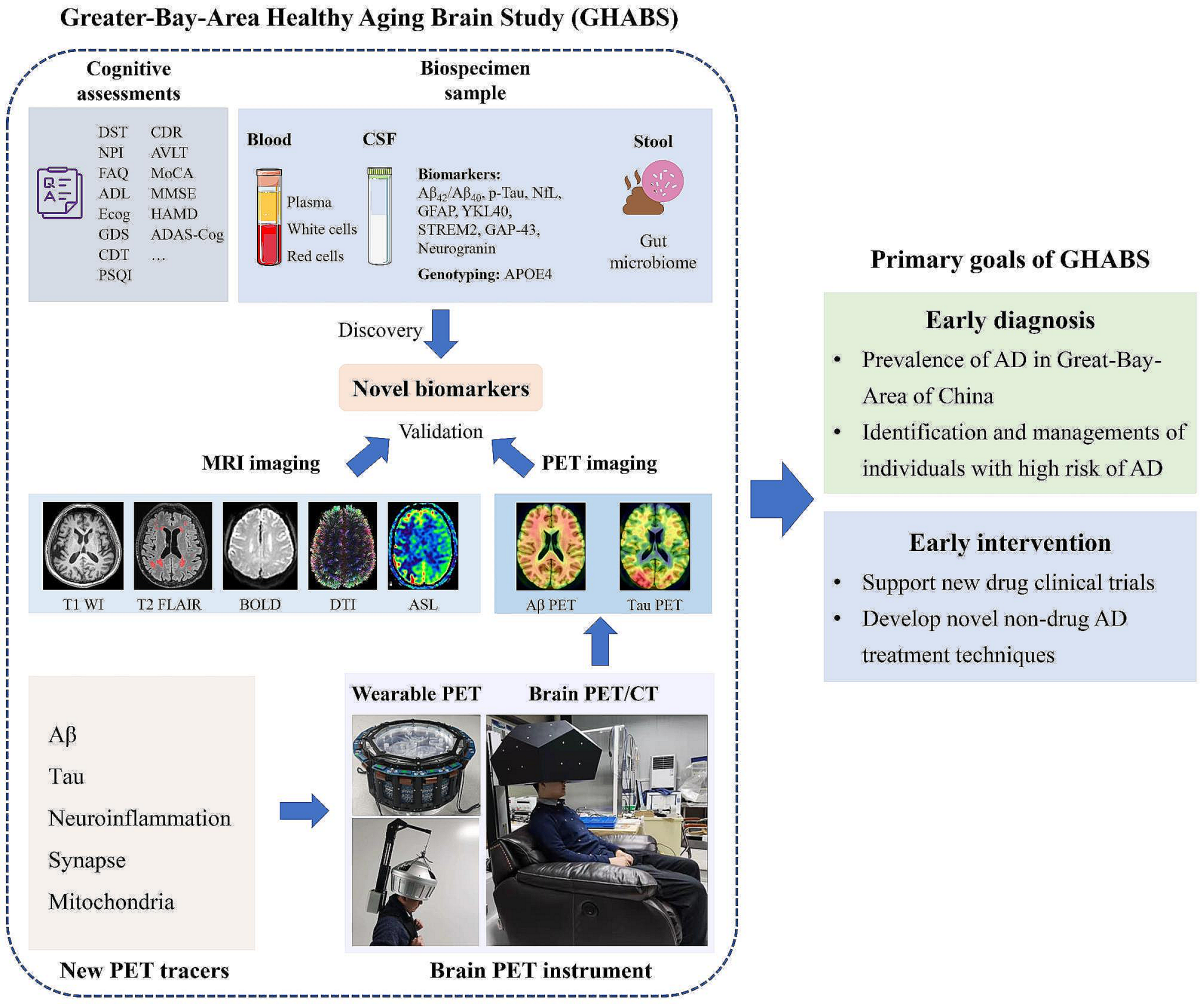
The general design and goals of the Greater-Bay-Area Healthy Aging Brain Study cohort

## Discussion

There are approximately 47 million preclinical AD individuals, 39 million MCI patients, and 15 million dementia patients in China. In 2035, the older population aged 60 and above is expected to exceed 400 million, accounting for more than 30% of the total population. As long as there are no practical methods for the early detection and treatment of AD, the number of AD patients in China will continue to rise. To end this, the GHABS study was initiated in 2021. The GHABS study aims to investigate the prevalence and progression of AD in the Guangdong-Hong Kong-Macao Greater-Bay-Area of China. The ultimate goal is to develop novel biomarkers and neuroimaging approaches for early diagnosis of AD and support the early intervention of clinical trials in the Guangdong-Hong Kong-Macao Greater-Bay-Area (Fig. 5).

Following the standard protocol of ADNI [50], the GHABS is supposed to be a high-standard AD community cohort in the Guangdong-Hong Kong-Macao Greater-Bay-Area of China. GHABS referred to several well-established large-scale AD cohorts from Europe and the United States, such as the Mayo Clinical Study of Aging [51], the Harvard Aging Brain Study [52], the Swedish Biomarkers For Identifying Neurodegenerative Disorders Early and Reliably, the Amyloid Imaging to Prevent Alzheimer’s Disease [53], and the Australian Imaging, Biomarkers and Lifestyle Study [54]. Based on these databases, many breakthroughs have been made in AD-related gene loci, biomarker detection, PET molecular imaging, etc., which provide important support for screening target participants and therapeutic targets in AD clinical trials. Besides, GHABS also referred to several well-organized cohorts in China, such as the Beijing Aging Brain Rejuvenation Initiative [55], China Aging and Neurodegenerative Initiative [56], Chinese Alzheimer’s Biomarker and LifestylE [57], the Chongqing Ageing & Dementia Study (CADS) [58], Chinese Preclinical Alzheimer’s Disease Study (C-PAS) [59], the China Cognition and Ageing Study [60], the Taizhou Imaging Study [61], and Sino Longitudinal Study on Cognitive Decline [62]. Dr. Ying Han, the president of the “Pre-Alzheimer’s Disease Alliance of China,” is one of the principal investigators in GHABS. Dr. Han initiated the Pre-AD Alliance of China in 2017 [63] and led two AD cohorts focused on the population of SCD for early AD diagnosis and investigation, the SILCODE and Cross-Cultural Longitudinal Study on Cognitive Decline [64]. The design of GHABS will be updated by the researchers if necessary. GHABS closely follows the latest academic and industry developments in the field and tries to adapt and update protocols.

The primary goal of GHABS is to investigate the pathological characteristics, risk factors, protective indicators, and evolution of Aβ and tau pathologies in Guangdong-Hong Kong-Macao Greater-Bay-Area older adults. The specific studies are as follows: (1) summarize the incidence of preclinical AD (Aβ + CU) in the Guangdong-Hong Kong-Macao Greater-Bay-Area, and reveal the characteristics and progression of AD pathologies; (2) study the feasibility of plasma biomarkers for detecting early AD in older adults; (3) determine the risk factors related to abnormal changes in Aβ and tau proteins; (4) reveal the spatiotemporal patterns of cortical Aβ plaques, tau tangles aggregation, synapse loss, and neuroinflammation and their relations to brain atrophy and cognitive decline; (5) investigate the roles of neuroinflammation, synaptic loss, vascular diseases, myelination, metabolic dysfunction across the spectrum of AD. Hopefully, these studies based on the GHABS cohort may provide novel insights into the early diagnosis and intervention of AD in China and the AD community.

Compared to CSF biomarkers and PET imaging, plasma biomarkers are the most promising early screening technology for AD by considering the advantages of simple sampling, low traumatic, and cost [65]. Recently, plasma Aβ_42_/Aβ_40_ [66, 67], p-Tau_181_ [68], p-Tau_217_ [69], p-Tau_231_ [70], and GFAP [71] showed great potential in early diagnosis of AD. One of the primary goals of GHABS is to evaluate the performance of previously reported plasma biomarkers and further explore novel plasma biomarkers in China’s aging population. For example, the GHABS research group has investigated the characteristics of CSF GAP43 in different clinical and pathological stages of AD [11] based on the ADNI cohort and demonstrated that presynaptic dysfunction measured by CSF GAP43 occurs prior to AD typical neurodegeneration and predicts faster cognitive decline [12]. We are currently measuring GAP43 concentrations in plasma in the GHABS cohort and evaluating its suitability as a plasma synaptic biomarker of AD in the Chinese aging population.

The GHABS project also supports and fertilizes new diagnosis methods for early AD diagnosis from academics and pharmaceutical industries. GHABS has been committed to co-developing early diagnostic tools since its inception, including fluid biomarkers, new antibodies, brain PET instruments, and new PET tracers. In Shenzhen Bay Laboratory, Dr. Qiyu Peng’s group are dedicated to developing high-performance and low-cost brain PET/CT scanner and wearable brain PET/CT scanner [72]. The GHABS plans to support the clinical verification of the novel brain PET/CT instruments, as AD is one of the main neurodegenerative diseases that require a brain-dedicated PET/CT instrument. GHABS is also exploring adjusting the Aβ PET imaging protocols for clinical diagnosis by shortening the scanning time or reducing trace dose using brain PET/CT scanners with high spatial resolution and detection sensitivity. In the future, GHABS will also facilitate AD clinical trials.

In this study, we, for the first time, investigated the abnormal alterations of plasma Aβ_42_, plasma Aβ_42_/Aβ_40_, plasma p-Tau_181_, plasma GFAP, plasma NfL, Aβ PET, tau PET, hippocampal volume, and AD-signature cortical thickness across the different clinical stages of AD based on a Chinese aging cohort in Guangdong-Hong Kong-Macao Greater-Bay-Area. This showed that SCD individuals had significantly higher plasma p-Tau_181_ and plasma GFAP than the NC individuals, suggesting that SCD may be related to early increases in plasma p-Tau_181_ and plasma GFAP. In addition, CI individuals (MCI or dementia) had abnormal changes in plasma Aβ_42_/Aβ_40_, p-Tau_181_, and NfL, and Aβ PET, tau PET, hippocampal volume, and AD-signature cortical thickness compared to the CU (NC or SCD) individuals. Moreover, the frequencies of plasma Aβ_42_/Aβ_40_ positive and plasma p-Tau_181_ positive (NC; 5.9%, SCD: 8.2%, MCI: 25.3%, Dementia: 64.9%) and Aβ PET positivity (NC; 25.6%, SCD: 22.5%, MCI: 47.7%, Dementia: 89.3%) were reported for the first time. The overall Aβ-PET positivity rates of NC, MCI, and dementia in C-PAS, an eastern China cohort, were 26.9%, 44.5%, and 85.8%, respectively [73], which was similar to the findings of the GHABS cohort. In general, the frequencies of Aβ PET positivity were in accordance with previous reports [74–76].

According to the latest NIA-AA research framework proposed by Jack and colleagues in AAIC 2023, it has been suggested to use a combination of plasma Aβ_42_/Aβ_40_ and plasma p-Tau rather than them alone to identify individuals with a high risk of AD. However, our findings indicate that using plasma Aβ_42_/Aβ_40_ positive and plasma p-Tau_181_ positive (A+/T+) may be only able to capture 23.0%, 36.4%, 53.4%, and 72.7% of Aβ PET positivity in NC, SCD, MCI, and dementia individuals respectively. Considering using A-/T + and A+/T- defined by plasma Aβ_42_/Aβ_40_ and plasma p-Tau_181_ in addition to A+/T + to identify individuals with a high risk of AD, the frequencies of abnormal plasma biomarkers increased by 46.2%, 44.4%, 73.6%, and 93.0% for NC, SCD, MCI, and Dementia respectively. Future investigation is required to determine whether A-/T + or A+/T- individuals defined by plasma Aβ_42_/Aβ_40_ and plasma p-Tau_181_ have evidence of Aβ plaques, tau tangles, brain atrophy, and cognitive decline.

In summary, we adapt the standard ADNI protocols to collect cognitive assessments, fluid biomarkers, and neuroimaging data to create a community-based observable AD cohort in the Guangdong-Hong Kong-Macao Greater Bay Area of China. The GHABS cohort is expected to identify novel biomarkers and neuroimaging techniques for early detection, determine the appropriate time window for AD intervention, and explore AD’s pathological features and progression patterns, especially during the asymptomatic stage of AD in South China’s aging population. We reported for the first time the pathophysiology characterization of plasma biomarkers, Aβ PET, tau PET, hippocampal atrophy, and AD-signature cortical thinning, as well as the prevalence of Aβ PET positivity in a Chinese community aging cohort in the Guangdong-Hong Kong-Macao Greater Bay Area of China. These findings provide important guidance towards designing standard AD community cohorts in South China and offer novel insights into understanding the characteristics of abnormal AD pathologies changes in South China’s older population.

### Electronic supplementary material

Below is the link to the electronic supplementary material.

Supplementary Material 1

## Data Availability

The data used in the current study were obtained from the GHABS cohort. Derived data is available from the corresponding author on request by any qualified investigator subject to a data use agreement.

## Abbreviations

AD: Alzheimer’s disease
ADAS-Cog: Alzheimer’s Disease Assessment Scale–Cognitive Subscale
ADL: Activity of Daily Living Scale
ADNI: Alzheimer’s Disease Neuroimaging Initiative
AFT: Animal Verbal Fluency Test
ASL: Arterial Spin Label
AVLT: Auditory Verbal Learning Test
Aβ: β-amyloid
BOLD: Blood Oxygen Level Dependent
CBF: Cerebral Blood Flow
CCI: Cognitive Change Index
CDR: Clinical Dementia Rating
CDT: Clock Drawing Test
CI: Cognitive Impaired
ci: confidence interval
CSF: Cerebrospinal Fluid
CU: Cognitively Unimpaired
CV: coefficient of variation
DST: Digit Span Test
DTI: Diffusion Tensor Imaging
Ecog: Everyday cognition
ELISA: Enzyme-linked Immunosorbent Assay
EPI: Echo-planar Imaging
ESS: Epworth Sleepiness Scale
FAQ: Function Activities Questionnaire
FBP: [^18^F]-florbetapir
FLAIR: Fluid-attenuated Inversion Recovery
fMRI: functional MRI
FSP: [^18^F]D3FSP
FTP: [^18^F]-flortaucipir
FWHM: Full-width at Half Maximum
GAP43: Growth-associated Protein-43
GDS: Geriatric Depression Scale
GFAP: Glial Fibrillary Acidic Protein
GHABS: Greater-Bay-Area Healthy Aging Brain Study
High Res Hippo: High Resolution Hippocampus
IQR: Interquartile Range
MCI: Mild Cognitive Impairment
MMSE: Mini-mental State Examination
MoCA-Basic: Montreal Cognitive Assessment Basic
MRI: Magnetic Resonance Imaging
NfL: Neurofilament Light
NPI: Neuropsychiatric Inventory
OR: Odds Ratio
pCASL: 3D pseudo-continuous Arterial Spin Labeling
PDGFR-β: Platelet-derived Growth Factor Receptorβ
PET: Positron Emission Tomography
PSQI: Pittsburgh Sleep Quality Index
p-Tau: Phosphorylated Tau
RBDSQ: REM Sleep Behavior Disorder Screening Questionnaire
rHCV: residual Hippocampal Volume
ROC: Receiver Operating Characteristic Curve
ROI: Region of Interest
SCD: Subjective Cognitive Decline
SDMT: Symbol Digit Modalities Test
SILCODE: Sino Longitudinal Study on Cognitive Decline
SNAP25: Synaptosome Associated Protein 25
sTREM2: Soluble Triggering Receptor Expressed on Myeloid Cells 2
STT: Shape Trail Test
SUVR: Standardized Uptake Value Ratio
SWI: Susceptibility Weighted Imaging
WMH: White-matter Hyperintensity
