# Supplementary material for: Pathophysiology characterization of Alzheimer’s disease in South China’s aging population: for the Greater-Bay-Area Healthy Aging Brain Study (GHABS)
Source: Alzheimers Res Ther. 2024 Apr 16;16:84. doi: 10.1186/s13195-024-01458-z (PMC11020808; doi:10.1186/s13195-024-01458-z)
Supplement: Supplementary file 1 — Supplementary Material 1 [file 13195_2024_1458_MOESM1_ESM.pdf]

Pathophysiology characterization of Alzheimer's disease in South China's Aging Population: for the Greater-Bay-Area Healthy Aging Brain Study (GHABS)

Zhen Liu[1#], Dai Shi[2 #], Yue Cai[1], Anqi Li[1], Guoyu Lan[1], Pan Sun[1], Lin Liu[1], Yalin Zhu[1], Jie Yang[1], Yajing Zhou[1], Lizhi Guo[1], Laihong Zhang[1], Shuqing Deng[1], Shuda Chen[2], Xianfeng Yu[3], Xuhui Chen[1, 4], Ruiyue Zhao[5], Qingyong Wang[6], Pengcheng Ran[7], Linsen Xu[8], Liemin Zhou[2], Kun Sun[9], Xinlu Wang[5], Qiyu Peng[1], Ying Han[1, 3, 10-12], Tengfei Guo[1,13,*]

[1] *Institute of Biomedical Engineering, Shenzhen Bay Laboratory, Shenzhen, 518132, China*

[2] *Neurology Medicine Center, The Seventh Affiliated Hospital, Sun Yat-sen University, Shenzhen, 518000, China*

[3] *Department of Neurology, Xuanwu Hospital of Capital Medical University, Beijing, 100053, China*

[4] *Department of Neurology, Peking University Shenzhen Hospital, Shenzhen, 518000, China*

[5] *Department of Nuclear Medicine, The First Affiliated Hospital, Guangzhou Medical University, Guangzhou, 510120, China*

[6] *Department of Neurology, University of Chinese Academy of Sciences-Shenzhen Hospital, Shenzhen, 518107, China*

[7] *Department of Nuclear Medicine, Guangdong Hospital of Traditional Chinese Medicine, Guangzhou, 510120, China*

[8] *Department of Medical Imaging, University of Chinese Academy of Sciences-Shenzhen Hospital, Shenzhen, 518106, China*

[9] *Institute of Cancer Research, Shenzhen Bay Laboratory, Shenzhen, 518132, China*

[10] *School of Biomedical Engineering, Hainan University, Haikou, 570228, China*

[11] *Center of Alzheimer's Disease, Beijing Institute for Brain Disorders, Beijing, 100053, China*

[12] *National Clinical Research Center for Geriatric Diseases, Beijing, 100053, China*

[13] *Institute of Biomedical Engineering, Peking University Shenzhen Graduate School, Shenzhen 518055, China.*

[#] **Equal contribution**

*** Corresponding author:**

Tengfei Guo, Ph.D., Institute of Biomedical Engineering,

Shenzhen Bay Laboratory, No.5 Kelian Road, Shenzhen, 518132, China.

Phone: 86-0755-2684-9264; E-mail address: tengfei.guo@pku.edu.cn

ORCID: 0000-0003-2982-0865

Supplementary Material

The participants' recruiting flowchart 3

Inclusion and exclusion criteria 4

Imaging analysis 11

Cutoffs of Aβ PET FSP SUVR 15

Cutoffs of plasma Aβ42/Aβ40 and plasma p-Tau181 16

Comparisons of plasma Aβ42 among different clinical stages 20

The participants' recruiting flowchart

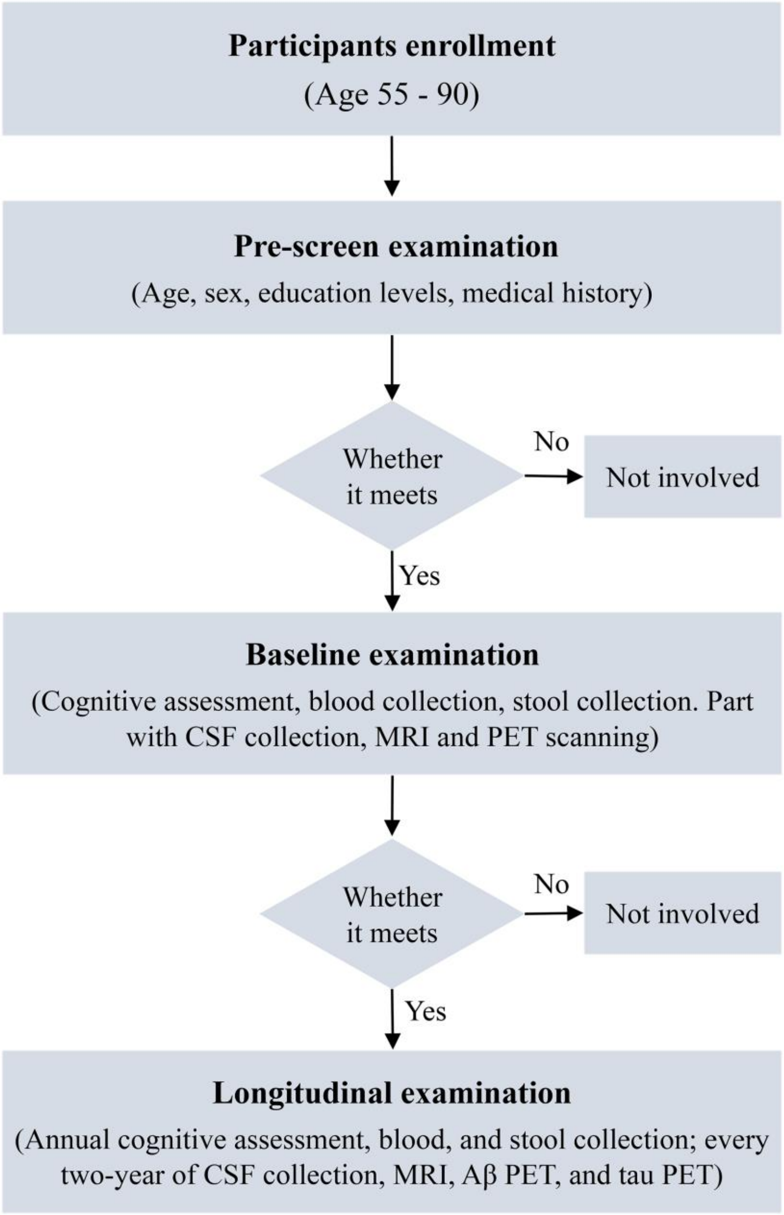

Supplemental Figure 1*. The scheme of enrolment and follow-up of participants in the Greater-Bay-Area Healthy Aging Brain Study (GHABS).*

Inclusion and exclusion criteria

Briefly, the inclusion criteria of GHABS are as follows: (1) adults between the ages of 55 and 90 and speak Mandarin fluently (notably, individuals below 60 years old should have a family dementia history and meet the criteria of subjective cognitive decline (SCD)[1]); (2) the score on the Geriatric Depression Scale (GDS) is less than 6 points; (3) visual and auditory acuity is sufficient for neuropsychological testing (Including normal corrected vision and hearing); (4) participants are not pregnant, lactating, or have reproductive potential (that is, women must be two years after menopause or undergo sterilization surgery); (5) a modified version of the Hachinski Ischemic scores less than or equal to 4; (6) have completed primary school (6 years of education) or have good work experience (sufficient to rule out mental retardation). Individuals with an infection, infarction, or other focal lesions or multiple lacunes or lacunes in critical memory structures and who do not meet the MRI scanning requirements are excluded from the GHABS study. More details of the inclusion and exclusion criteria can be found in the supplemental materials.

This project intends to recruit men and women between the ages of 55 and 90. The inclusion criteria are as listed:

- Age 55-90 years old (including 55 and 90 years old). Notably, individuals below 60 years old are required to have a family dementia history and meet the criteria of subjective cognitive decline (SCD), while people with family history of autosomal dominant or other familial AD are not limited by age.
- The score of the Geriatric Depression Scale (GDS) is less than 6 points.
- There is a caregiver who can maintain at least 10 hours of contact per week and can accompany volunteers to the test site for testing.
- Visual and auditory acuity is sufficient for neuropsychological testing. (Including normal corrected vision and hearing)
- Be in good health and are expected to be free of disease interference during the study.

- Volunteers are not pregnant, lactating or have reproductive potential (that is, women must be two years after menopause or undergo sterilization surgery).
- Willingness and ability to participate in longitudinal imaging studies.
- A modified version of the Hachinski Ischemic scores less than or equal to 4.
- Have completed grade 6 education or have good work experience (sufficient to rule out mental retardation).
- Must be able to speak Mandarin fluently.
- Willing to undergo multiple 3T MRI scans and at least two PET scans.
- Agree to collect blood for genomic analysis (including GWAS sequencing and other analyses), AD risk and protective genes such as apolipoprotein E (APOE), klotho, etc., and biological sample storage.
- Agree to collect blood for biomarker detection.
- Agree to share genomic data and biomarker samples.

The exclusion criteria are as follows:

- MRI brain scan screening reveals infection, infarction or other focal lesions or multiple lacunes or lacunes in key memory structures.
- Any volunteers who do not meet the MRI scan requirements, including having a cardiac pacemaker, eyes, skin or metal fragments or foreign bodies in the body.
- Severe depression, bipolar affective disorder described in DSM-IV in the past year.
- Psychotic features, agitation or behavioral problems that may lead to difficulty complying with the protocol content in the past 3 months.
- Currently using medication to treat obsessive-compulsive disorder or attention deficit disorder.
- History of schizophrenia (meeting DSM-IV criteria).
- History of alcohol or drug abuse or dependence within the past 2 years (meeting DSM-IV criteria).

- Any major systemic disease or unstable physical condition that may make longitudinal research difficult.
- Clinically significant abnormalities of B12 or TFTs may interfere with the study, low B12 will be excluded.
- Currently using certain psychoactive medications (e.g., certain antidepressants, neuro-depressants, chronic anxiolytics, or sedative-hypnotics). Currently using warfarin or other anticoagulants such as dabigatran, rivaroxaban, and apixaban (except lumbar puncture).
- Use of prohibited drugs.
- Simultaneously participating in other clinical studies involving neuropsychiatry.

Demographic data of Greater Bay Area of China

The demographic characteristics of the Greater Bay Area in China, focusing on city-specific data, are obtained from official statistics. This information is concisely presented in Supplemental Table 1. Given that the GHABS cohort is community-based, with a majority of participants hailing from Shenzhen, we delved deeper into the sex, education, and risk factor distribution within this cohort. Our findings are compiled in Supplemental Table 2. In addition, we scoured relevant literature to identify and extract key data points that are directly comparable with our study. These data are showcased in Supplemental Table 3, along with references to the corresponding literature[2][3].

From the comparison of data between the Supplemental Table 2 and 3, the prevalence of cognitive impairment (CI) in the statistical population is quite similar between the GHABS cohort (30.64%) and the literature (33.30%). Both studies exhibit a higher proportion of female participants. Notably, the GHABS cohort has a lower percentage of females within the CI group. A notable difference is observed in the percentage of APOE-ε4 carriers, with the GHABS cohort showing a significantly higher proportion (43.06%) compared to the literature (20.77%). In terms of education, the GHABS population demonstrates a higher level of educational attainment, with a greater number of participants possessing a high school diploma or higher. When examining the rates of hypertension, we found that the GHABS cohort has a lower prevalence in both the cognitively unimpaired (CU) and CI groups compared to the literature. Conversely, the diabetes rate is higher in the CU group of the GHABS cohort but lower in the CI group. Unfortunately, a direct comparison of hyperlipidemia rates between the two studies could not be made, as the literature reported data on dyslipidemia instead.

Supplemental Table 1 Demographic data of Greater Bay Area of China by city

Region	Resident population		Sex		Age		Education			
	Population (2020)	Proportion (%)	Male (%)	Female (%)	60 and above (%)	65 and above (%)	College (%)	High school (%)	Middle school (%)	Elementary school (%)
Guangzhou	18676605	0.22	52.83	47.17	11.41	7.82	27.28	22.01	29.48	13.78
Shenzhen	17560061	0.21	55.04	44.96	5.36	3.22	28.85	20.70	31.22	11.51
Zhuhai	2439585	0.03	53.46	46.54	10	6.64	25.75	21.58	28.90	15.36
Foshan	9498863	0.11	54.36	45.64	10.52	7.35	16.14	20.56	36.32	18.68
Huizhou	6042852	0.07	53.77	46.23	10.05	6.83	12.32	16.22	40.20	21.52
Dongguan	10466625	0.12	56.53	43.47	5.47	3.54	13.24	22.28	42.22	15.71
Zhongshan	4418060	0.05	53.94	46.06	8.87	5.98	13.36	19.60	38.57	20.02
Jiangmen	4798090	0.06	51.80	48.20	18.26	13.01	11.84	20.65	36.53	22.84
Zhaoqing	2141295	0.03	52.05	47.95	16.41	11.81	8.79	14.86	39.90	25.24
Hong Kong#	7426700	0.09	45.60	54.40	27.40	19.10	34.30	33.30	14.50	18.00
Macao*	682070	0.01	46.96	53.04	18.96	12.14	30.47	28.62	17.89	20.30

Demographic data of nine cities in Guangdong province are from: 1) Guang Dong Statistical Yearbook. China Statistics Press, 2021. 2) https://www.stats.gov.cn/sj/pcsj/

Data are from https://www.censtatd.gov.hk/en/scode150.html

* Data are from Demographic Statistics of 2021. https://www.dsec.gov.mo/en-US/Statistic?id=101

Supplemental Table 2 Demographic data of GHABS

	CU group	(%)	CI group	(%)
No., %	326	69.36	144	30.64
Female (No., %)	222	68.10	79	54.86
APOE-ε4 (No., %)	60	18.40	62	43.06
None (illiterate)	1	0.31	3	2.08
Elementary school	9	2.76	14	9.72
High school or above	216	66.26	58	40.28
Hypertension (No., %)	65	19.94	26	18.06
Diabetes (No., %)	28	8.59	14	9.72
Hyperlipidemia (No., %)	86	26.38	17	11.81

Supplemental Table 3 Demographic data of the Greater Bay Area (GBA) of China from literature

	Control group	(%)	ADOD group	(%)
No., %	2600	66.67	1300	33.33
Female	1760	67.70	880	67.70
APOE-ε4 (No., %)	477	18.35	270	20.77
None (illiterate)	4	0.15	795	61.15
Elementary school	1064	40.92	426	32.77
High school or above	229	8.81	79	6.08
Hypertension	910	35.00	529	40.69
Diabetes	74	2.85	166	12.77
Dyslipidemia	706	27.15	324	24.92

The demographic statistic data was collected from 2016-2018. Data is derived from: 1) Weekes B, Carthery-Goulart MT. Intervention and Prevention of Dementia in the Greater Bay Area (GBA) of China. American Journal of Alzheimer's Disease & Other Dementias® 2023, 38: 15333175231211097. 2) Qi SG, Wang ZH, Wei CB, Yang Z, Zhu XQ. [Case-control study on the influencing factors related to cognitive impairment in the elderly population of China]. Zhonghua Yu Fang Yi Xue Za Zhi 2018, 52(9): 926-931.

Current participants in GHABS

The primary affiliation of the GHABS cohort was located in Shenzhen, which is the third-largest city in China, followed by the first-largest city Shanghai and second-largest city Beijing. As shown in Supplemental Figure 2, the majority (521/627) of GHABS participants were located in Shenzhen, followed by Guangzhou (51/627), Huizhou (19/627), Dongguan (11/627), Jiangmen (4/627), Foshan (3/627), Zhongshan (2/627), Zhuhai (2/627), Zhaoqing (2/627), and Macao (1/627). Although the GHABS initiative has yet to gain widespread recognition in certain areas, participant convenience is a notable challenge, especially for those residing in cities geographically distant from Shenzhen. We are still trying to address this issue by recruiting more participants from other cities within the Greater-Bay-Area outside Shenzhen.

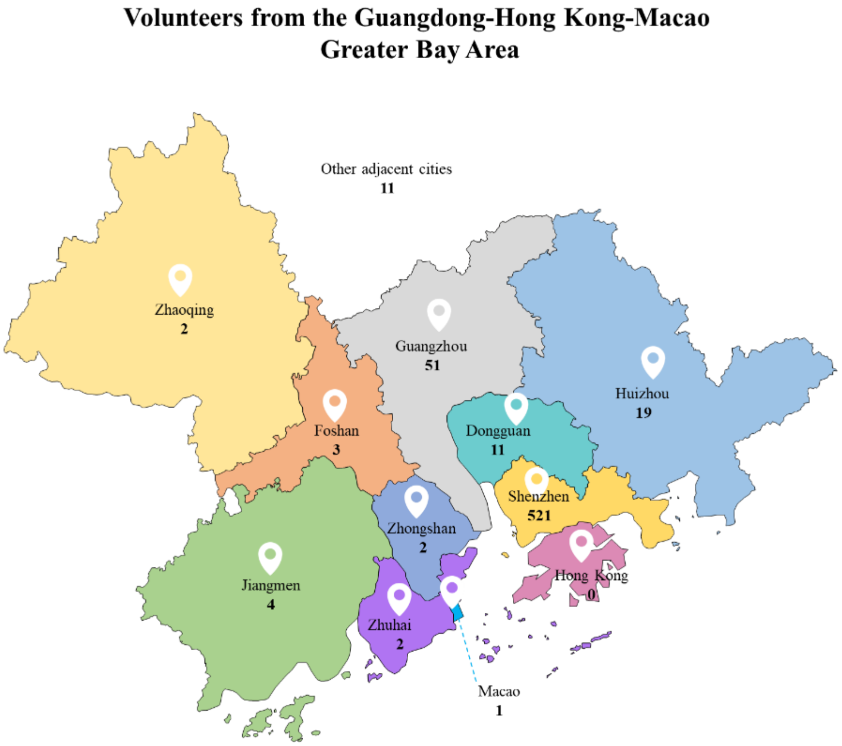

Supplemental Figure 2. *Map show of the older adults that from different cities of the Greater-Bay-Area in China*

Imaging analysis

The general process of resting-state data preprocessing and brain functional connectivity construction are outlined in the third column of Figure 2 in the main text. Preprocessing of resting-state image data was performed using the SPM12 software package in Matlab. The preprocessing steps are as follows: 1) first, the Dicom format of the original image data was converted to nii format using the built-in Dcm2nii module in SPM12; 2) the first 10 images of each data were removed to reduce the interference caused by magnetic field instability; 3) temporal layer correction; 4) head motion correction, with a maximum translation of less than 1mm in the X, Y, and Z axes and an average rotation angle of less than 1° around these three directions in this study; 5) resting-state fMRI data underwent the geometry distortion correction based on the fieldmap using a similar method in the ASL imaging processing; 6) spatial normalization: GHABS used individual T1 and standard space T1 structural images for joint segmentation-based spatial normalization; 7) spatial smoothing: this study used a Gaussian kernel function with a FWHM of 6mm to smooth the images to meet the distribution requirements of Gaussian random fields; 8) band-pass filtering was used with a frequency range of 0.01 Hz-0.1 Hz. Based on the time series of AAL-116 brain regions obtained from the preprocessed data, a correlation analysis was performed to obtain a 116×116 time series correlation matrix. The Fisher's Z transformation was applied to the 116×116 time series correlation matrix to obtain the whole-brain functional connectivity matrix.

The general process of diffusion MRI are outlined in the fourth column of Figure 2 in the main text. Diffusion-weighted data were denoised and corrected for Gibbs ring using Mrtrix3 (V3.0.3)[4], and then corrections were applied for head motion, eddy current and EPI susceptibility distortion using FSL (V6.0.3)[5]. Next, a brain mask was constructed using Brain Extraction Tool (BET)[6], and diffusion tensor model was reconstructed within the brain mask for estimation of fractional anisotropy (FA) parameter map with b=1000/mm^2. For the assessment of neurite orientation dispersion and density imaging (NODDI)[7], Accelerated Microstructure Imaging via Convex Optimization (AMICO)[8] was employed with b=1000 and 2000 s/mm^2. NODDI parameter maps, including neurite density index (NDI),

orientation dispersion index (ODI), and isotropic volume fraction (ISO) were estimated. Subsequently, region of ROI-based analysis was performed respectively for FA, NDI, ODI and ISO, by non-linearly warping the JHU “Eve” WM atlas[9] to each participant. Furthermore, deterministic fiber tracking based on FA was conducted using DSI-studio[10] to construct a whole-brain structural connectivity map. The aforementioned functional connectivity brain region AAL-116 template was integrated with structural connectivity results, yielding the structural connectivity matrix.

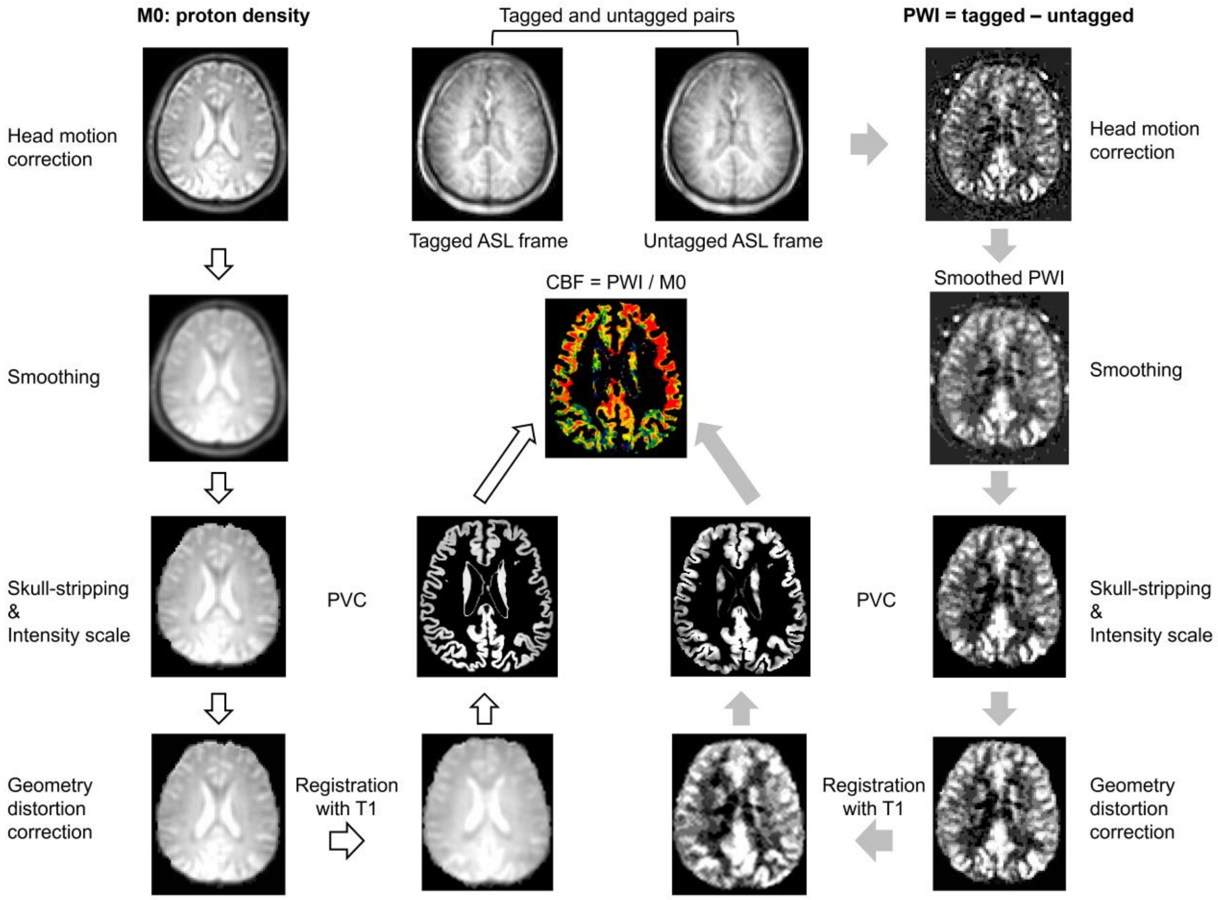

***Supplemental Figure 3**. Schematic diagram of ASL processing pipeline.*

The general imaging processing pipeline of ASL involved several steps (Supplemental Figure 3). First, ASL frames underwent head motion correction using SPM12. Second, the perfusion images were obtained by subtracting the mean of tagged from untagged ASL images, and both perfusion and proton

density images were smoothed using a Gaussian kernel with a full width at FWHM of 4mm. To correct EPI geometry distortion, a fieldmap with units of radians per second was obtained from raw fieldmap images using FSL (V6.0.3). The calculated fieldmap and structural T1 images were then used to correct for geometry distortion for perfusion and proton density images. Subsequently, perfusion and proton density images were co-registered with the T1 images, and underwent partial volume correction[11]. Finally, the quantitative CBF image was computed based on perfusion and proton density images[12], and regional mean CBF of 68 FreeSurfer-defined ROIs (regions of interest) defined by FreeSurfer were extracted in the native T1 space.

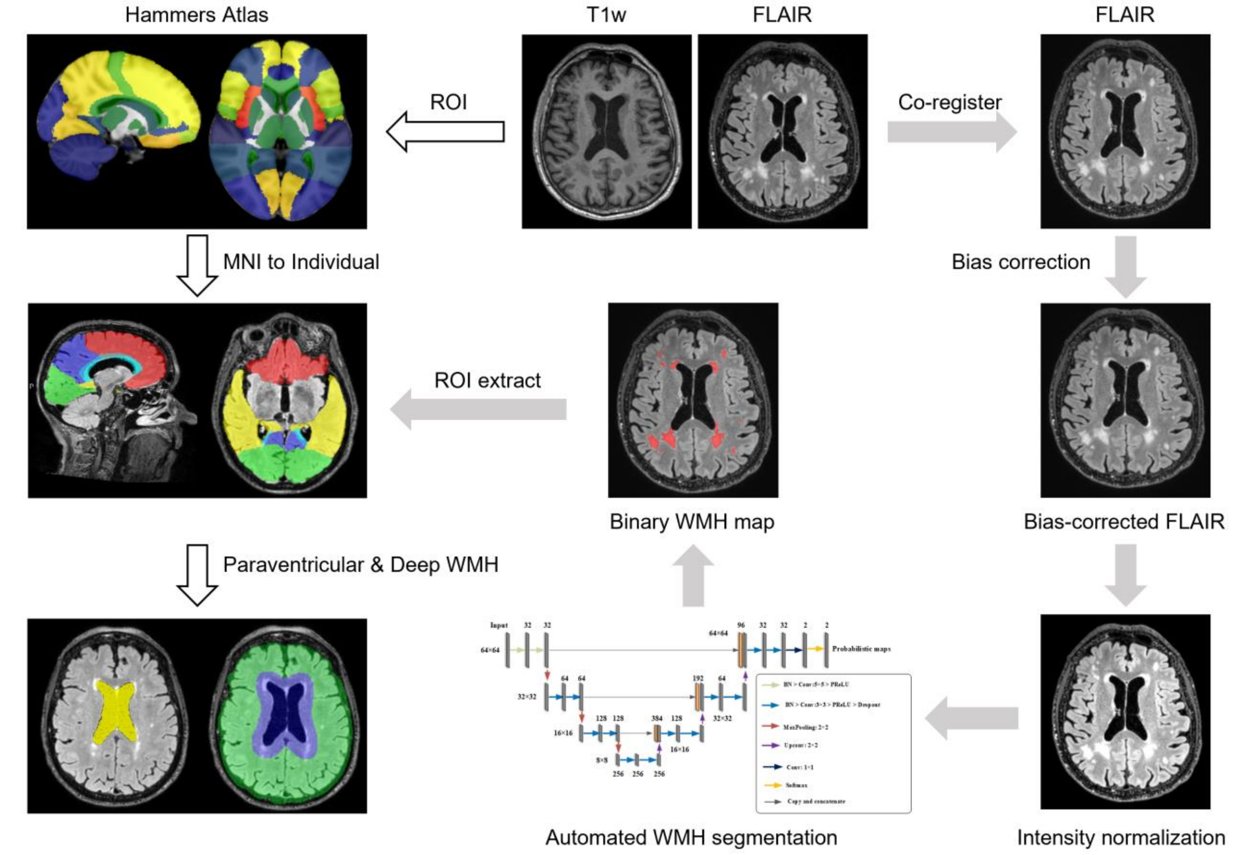

Supplemental Figure 4. *Schematic diagram of T2 FLAIR processing pipeline.*

The general imaging processing pipeline of T2 FLAIR are outlined in Supplemental Figure 4. The white-matter hyperintensity (WMH) segmentation was processed using a custom pipeline developed by our lab based on the T2 FLAIR (Fluid-Attenuated Inversion Recovery) images. Briefly, T1 structural image was segmented using Freesurfer (v7.2.0) to derive the bias field corrected T1 images and CSF mask, and the original T2 FLAIR image was co-registered to the corrected T1 image. Then, an anatomical-based MRI deep learning model (^{A}U-Net) was used with T2 FLAIR image as the input[13], and a binary WMH was obtained. The resulting WMH mask was further corrected for false positives in the choroid plexus of the lateral ventricle based on the CSF mask, and were manually edited by two senior physicians to ensure consistency and accuracy. Finally, the frontal, parietal, occipital, temporal atlas from the Hammer template (Hammers atlas[14]) were transformed to individual T1 space, and the regional WMH volumes were then extracted.

Cutoffs of Aβ PET FSP SUVR

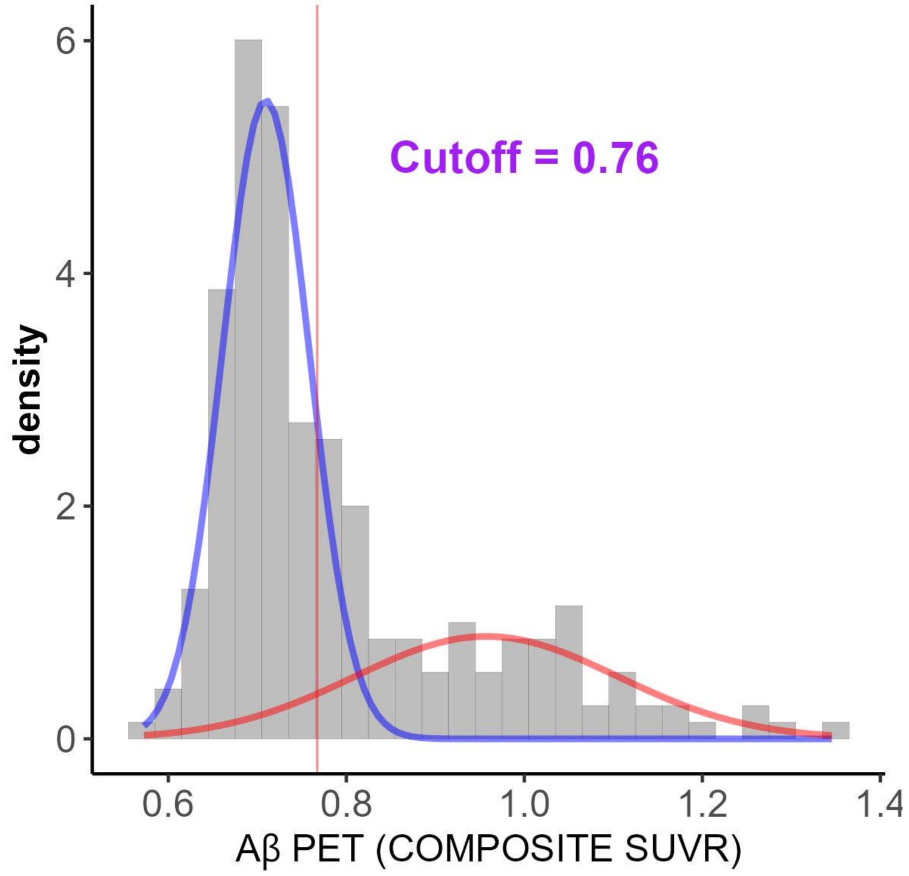

Supplemental Figure 5. *Estimates of two Gaussian distributions of low Aβ (blue curve) and high Aβ (red curve) for COMPOSITE FSP SUVRs of 233 individuals. The red dash vertical line reflects the Aβ+ threshold of COMPOSITE FSP SUVR 0.76, which corresponds to a 90% probability of belonging to the high Aβ distribution.*

Cutoffs of plasma Aβ42/Aβ40 and plasma p-Tau181

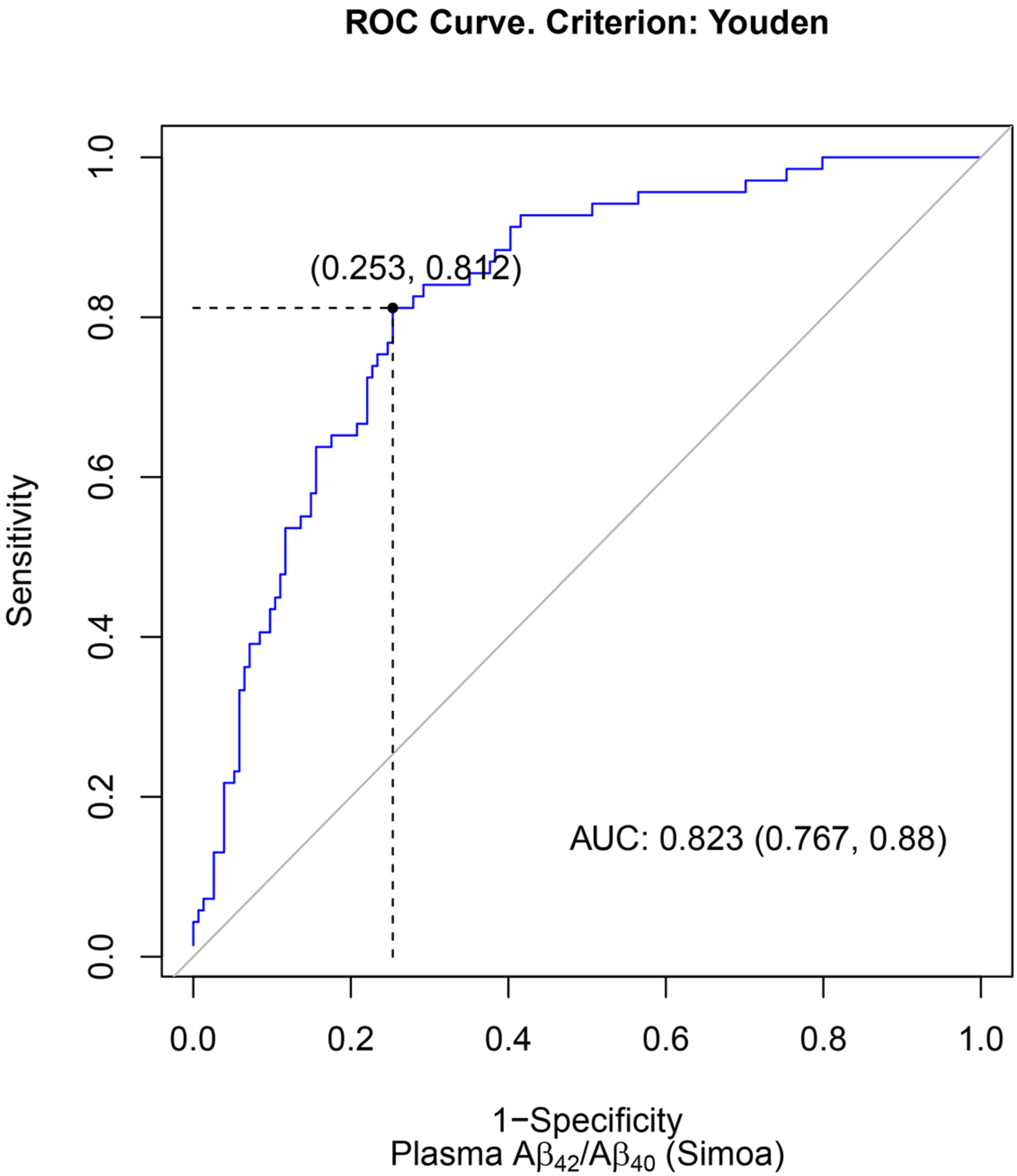

Supplemental figure 6. *The ROC analysis using the Youden index classifying 154 Aβ- cognitively unimpaired (CU) participants and 69 Aβ+ mild cognitive impairment (MCI) and dementia patients as the endpoint to define the cutoff ≤ 0.0609 for plasma* $A\beta_{42}/A\beta_{40}$ *ratio. AUC: 0.823 (95% CI, 0.767, 0.88).*

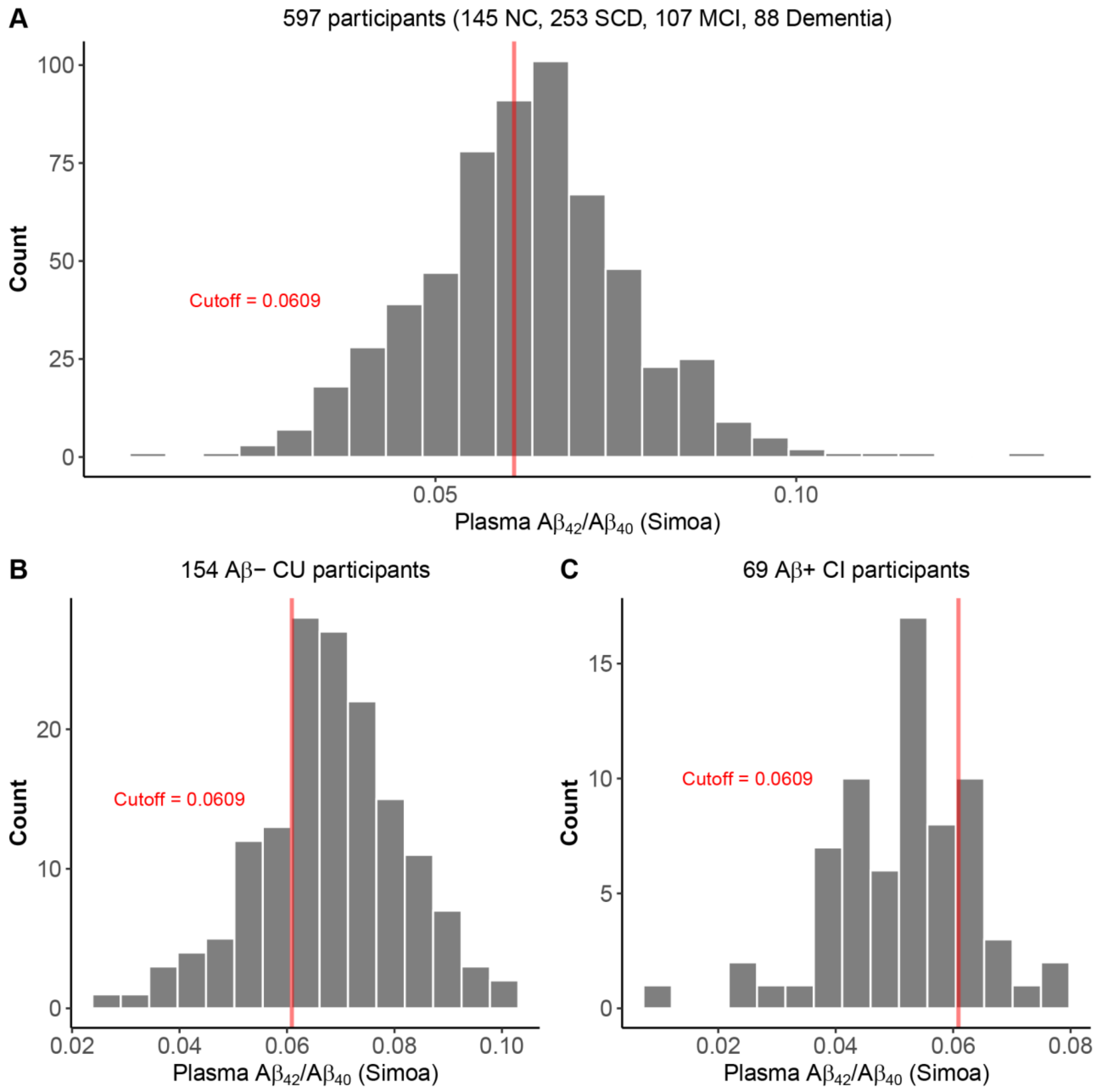

Supplemental figure 7. *Histograms of plasma $A\beta_{42}/A\beta_{40}$ for (A) all 597 GHABS participants, (B) 154 Aβ- GHABS CU participants and (C) 69 Aβ+ GHABS MCI and dementia patients. The red dotted line is the cutoff ≤0.0609 for the plasma $A\beta_{42}/A\beta_{40}$ ratio.*

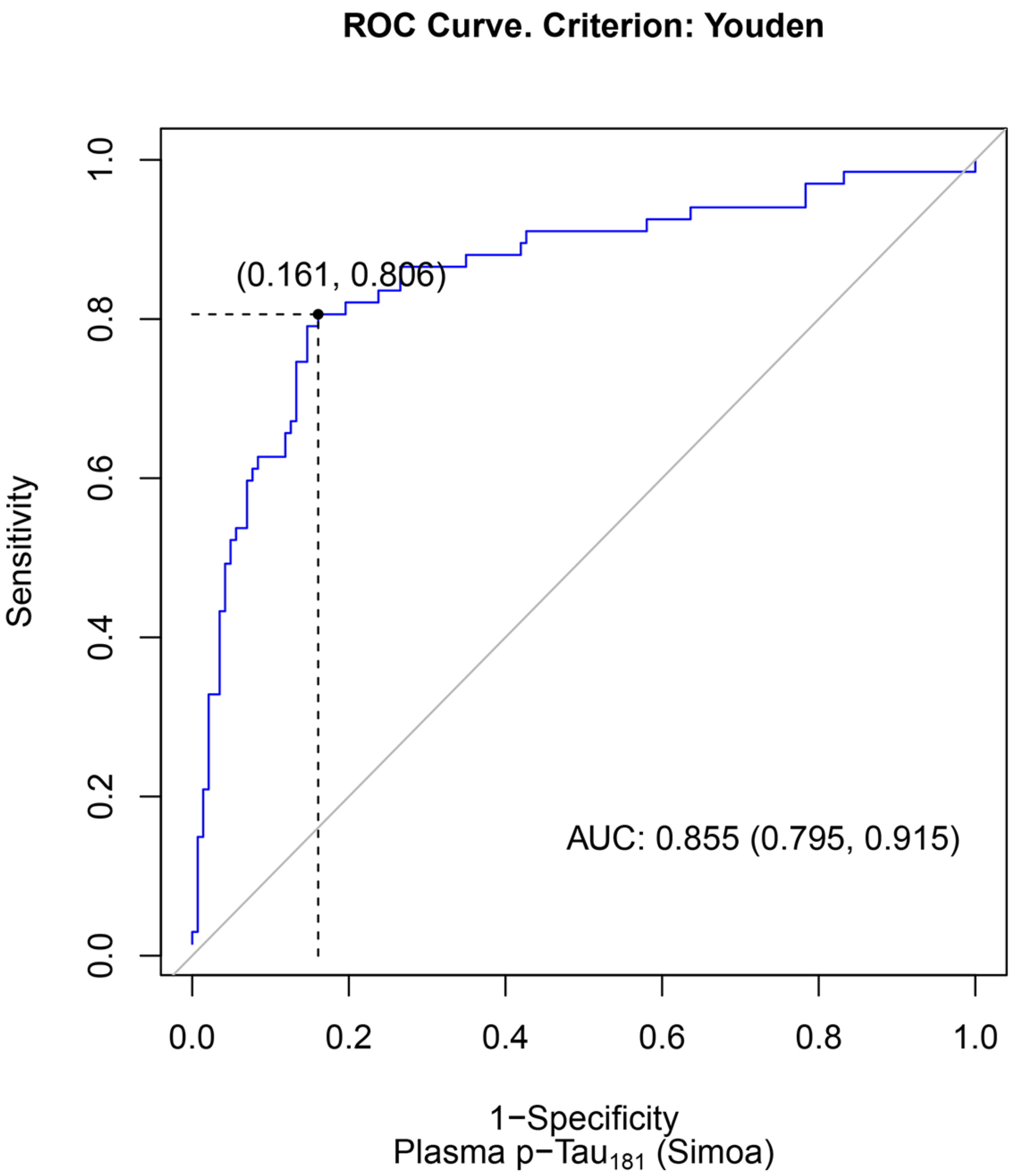

Supplemental figure 8. *The ROC analysis using the Youden index classifying 143 Aβ- GHABS CU participants and 67 Aβ+ GHABS MCI and dementia patients as the endpoint to define the cutoff ≥ 2.418 for plasma p-Tau*$_{181}$*. AUC: 0.855 (95% CI, 0.795, 0.915).*

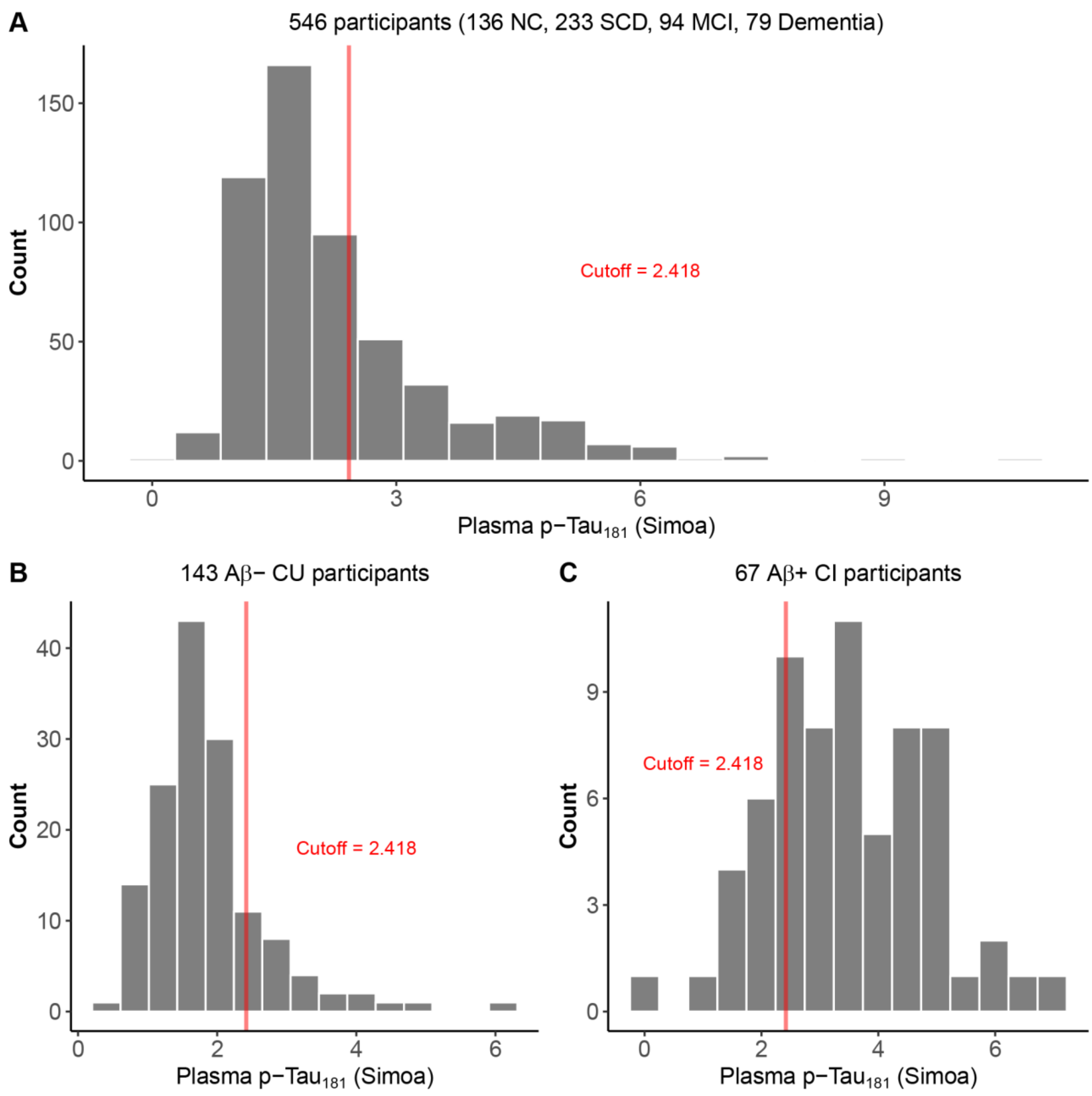

Supplemental figure 9. *Histograms of plasma p-Tau$_{181}$ for (A) all 545 GAHBS participants, (B) 143 Aβ- GHABS CU participants and (C) 67 Aβ+ GHABS MCI and dementia patients. Red dotted line is the 2.418 cutoff for the plasma p-Tau$_{181}$.*

Comparisons of plasma Aβ42 among different clinical stages

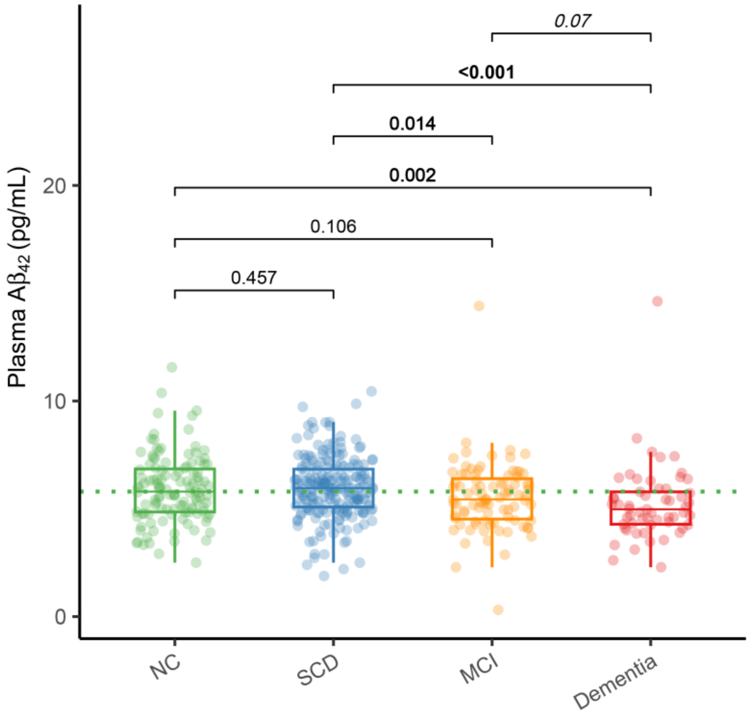

Supplemental figure 10. Comparisons of plasma Aβ42 among NC, SCD, MCI, and dementia groups.

References

1. Jessen F, Amariglio RE, Buckley RF, van der Flier WM, Han Y, Molinuevo JL, et al. The characterisation of subjective cognitive decline. Lancet Neurol. 2020;19:271–8.

2. Weekes B, Carthery-Goulart MT. Intervention and Prevention of Dementia in the Greater Bay Area (GBA) of China. Am J Alzheimer's Dis Other Dementias® [Internet]. 2023;38:15333175231211096. Available from: https://doi.org/10.1177/15333175231211097

3. Qi SG, Wang ZH, Wei CB, Yang Z, Zhu XQ. [Case-control study on the influencing factors related to cognitive impairment in the elderly population of China]. Zhonghua Yu Fang Yi Xue Za Zhi. 2018;52:926–31.

4. Tournier J-D, Smith R, Raffelt D, Tabbara R, Dhollander T, Pietsch M, et al. MRtrix3: A fast, flexible and open software framework for medical image processing and visualisation. Neuroimage. 2019;202:116137.

5. Jenkinson M, Beckmann CF, Behrens TEJ, Woolrich MW, Smith SM. FSL. Neuroimage. 2012;62:782–90.

6. Smith SM. Fast robust automated brain extraction. Hum Brain Mapp. 2002;17:143–55.

7. Zhang H, Schneider T, Wheeler-Kingshott CA, Alexander DC. NODDI: practical in vivo neurite orientation dispersion and density imaging of the human brain. Neuroimage. 2012;61:1000–16.

8. Daducci A, Canales-Rodríguez EJ, Zhang H, Dyrby TB, Alexander DC, Thiran J-P. Accelerated Microstructure Imaging via Convex Optimization (AMICO) from diffusion MRI data. Neuroimage. 2015;105:32–44.

9. Oishi K, Faria A, Jiang H, Li X, Akhter K, Zhang J, et al. Atlas-based whole brain white matter analysis using large deformation diffeomorphic metric mapping: application to normal elderly and Alzheimer's disease participants. Neuroimage. 2009;46:486–99.

10. Yeh F-C, Verstynen TD, Wang Y, Fernández-Miranda JC, Tseng W-YI. Deterministic diffusion fiber tracking improved by quantitative anisotropy. PLoS One. 2013;8:e80713.

11. Alsop DC, Detre JA, Golay X, Günther M, Hendrikse J, Hernandez-Garcia L, et al. Recommended implementation of arterial spin-labeled perfusion MRI for clinical applications: A consensus of the ISMRM perfusion study group and the European consortium for ASL in dementia. Magn Reson Med. 2015;73:102–16.

12. Kanetaka H, Matsuda H, Asada T, Ohnishi T, Yamashita F, Imabayashi E, et al. Effects of partial volume correction on discrimination between very early Alzheimer's dementia and controls using brain perfusion SPECT. Eur J Nucl Med Mol Imaging. 2004;31:975–80.

13. Liang L, Zhou P, Lu W, Guo X, Ye C, Lv H, et al. An anatomical knowledge-based MRI deep learning pipeline for white matter hyperintensity quantification associated with cognitive impairment. Comput Med imaging Graph Off J Comput Med Imaging Soc. 2021;89:101873.

14. Hammers A, Allom R, Koepp MJ, Free SL, Myers R, Lemieux L, et al. Three-dimensional maximum probability atlas of the human brain, with particular reference to the temporal lobe. Hum Brain Mapp. 2003;19:224–47.
